# Integrated multi-omics reveals lipid remodeling underlying population- and stage-associated aroma variation in chicken egg yolks

**DOI:** 10.1016/j.fochx.2026.104548

**Published:** 2026-10-01

**Authors:** Cui Ma, Jinsong Pi, Ailuan Pan, Yaru Chen, Ming Fu, Tao Huang, Hao Zhang, Yan Wu

**Affiliations:** a Institute of Animal Science and Veterinary Medicine, Hubei Academy of Agricultural Sciences, Wuhan 430064, China; b Key Laboratory of Animal Embryo Engineering and Molecular Breeding of Hubei Province, Wuhan, 430064, China

**Keywords:** Egg yolk aroma, Volatilomics, Lipid metabolic remodeling, Odor-relevant volatiles, Laying progression, Multi-omics

## Abstract

Egg yolk aroma is a key determinant of consumer acceptance, yet the metabolic basis of aroma variation across chicken breeds and laying stages remains unclear. Here, integrated volatilomics, lipidomics, and metabolomics were employed to investigate egg yolk aroma variation in four genetically distinct chicken populations at the onset-of-lay and peak-laying stages. A total of 265 volatile compounds were tentatively annotated. Literature odor thresholds were available for 93 compounds, allowing their OAVs to be calculated; among these, aldehydes, alcohols, ketones, and terpenoids were the predominant classes. Factorial analyses revealed that sampling stage was associated with broader molecular changes across the yolk volatilome, lipidome, and metabolome, whereas population-associated effects were more selective. At the onset of lay, population-associated variation involved phospholipid remodeling, LPC/LPA-related metabolism, and differences in odor-relevant 2-undecenal isomers. During peak laying, higher abundances of several lipid oxidation-derived aldehydes, including heptanal, nonanal, and (E)-2-octenal, were accompanied by extensive remodeling of glycerophospholipids, sphingolipids, and triacylglycerols, together with alterations in carbon metabolism, the pentose phosphate pathway, and glycerophospholipid metabolism. Integrated multi-omics analysis revealed coordinated associations among lipid remodeling, oxidation-sensitive lipid precursor pools, and odor-relevant volatile compounds. Collectively, these findings indicate that stage-associated metabolic remodeling represents a major feature of egg yolk aroma variation, while population-associated differences involve more selective changes in specific precursor pools. This study provides a metabolic framework for understanding population- and stage-associated variation in egg yolk aroma.

## Highlights

• Sampling stage was associated with broader changes in egg yolk aroma and metabolism.
• Population-associated differences involved selective volatile and metabolic features.
• Lipid oxidation-derived aldehydes varied markedly across the two sampling stages.
• Phospholipid remodeling was closely associated with odor-relevant lipid precursor pools.
• Chuqin No. 2 exhibited distinct metabolic features associated with yolk aroma variation.

## Graphical abstract

**Figure:**
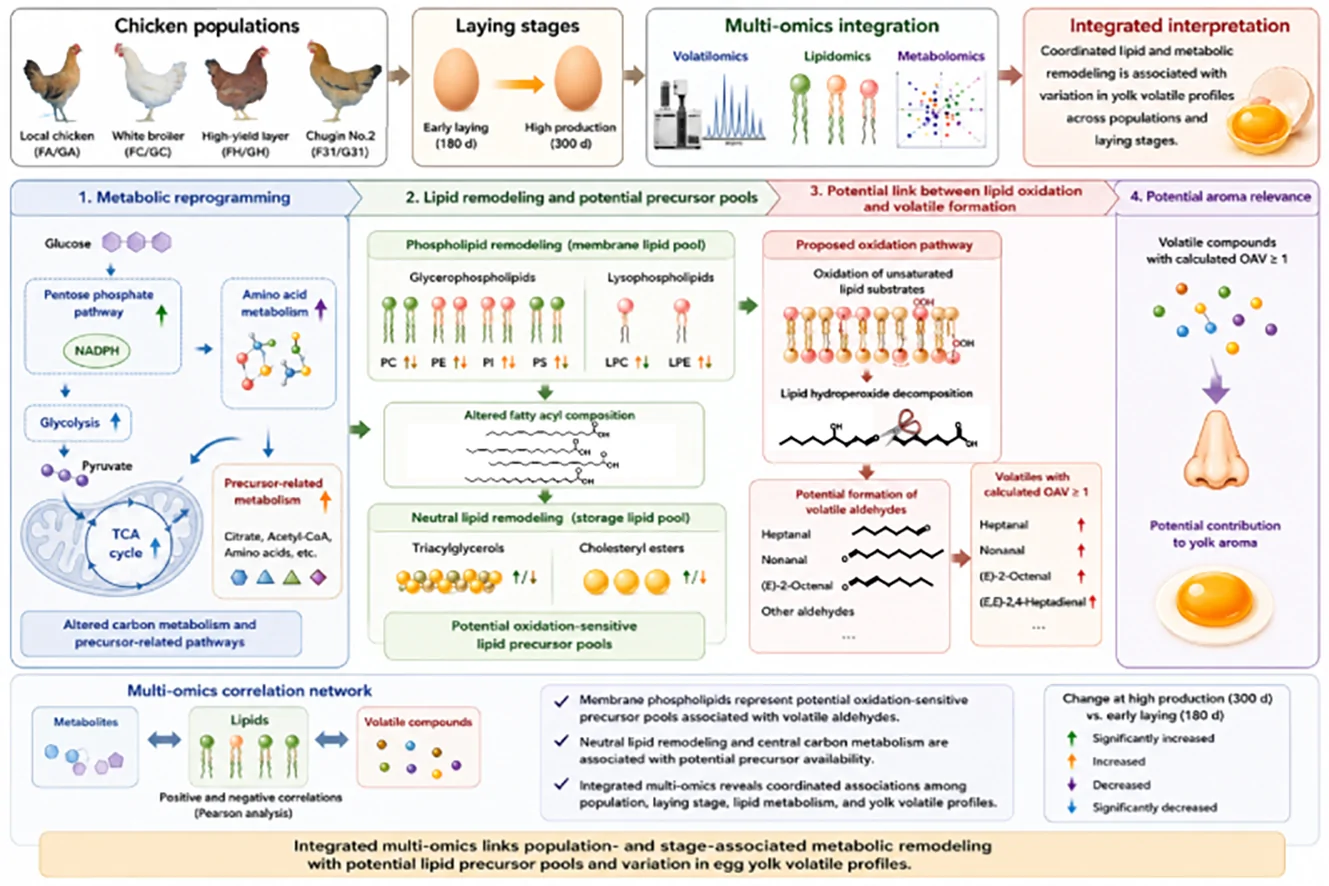

## 1 Introduction

Poultry eggs are widely consumed worldwide due to their balanced nutritional composition and high biological value, providing essential macronutrients and micronutrients in human diets (Lu et al., 2025). In addition to nutritional attributes, sensory quality has become an increasingly important criterion for evaluating egg quality, with flavor being a major factor influencing consumer acceptance and product differentiation. Among the egg components, the yolk plays a central role in flavor formation because of its high lipid content and abundance of potential volatile precursors. Lipid classes, including glycerolipids and phospholipids, provide substrates susceptible to oxidative transformation, leading to the generation of volatile compounds that contribute to characteristic egg aroma. Lipid oxidation, together with amino acid-derived reactions, represents an important biochemical pathway involved in the formation of odor-relevant volatile compounds in eggs (Ma et al., 2026). Therefore, variations in yolk composition, particularly in the abundance and distribution of lipid substrates, may influence volatile profiles and thereby contribute to differences in egg flavor.

Egg yolk flavor profiles are closely associated with the genetic background of poultry, which influences nutrient deposition, lipid metabolism, and fatty acid composition (Ma et al., 2026). Different breeding objectives have generated distinct metabolic characteristics among native chickens, commercial laying hens, and broilers. Native chickens generally exhibit unique flavor profiles, whereas commercial laying hens have been intensively selected for high egg production efficiency, and broilers have been primarily optimized for rapid growth performance and feed conversion rather than egg-related traits (Liu et al., 2024). Differences among these populations may be accompanied by variation in yolk lipid deposition and fatty acid composition, which can influence the abundance and distribution of lipid substrates potentially involved in volatile formation. However, previous studies have mainly focused on differences in fatty acid composition or volatile profiles, while the relationships among population-associated lipid remodeling, potential volatile precursor pools, and yolk flavor remain incompletely understood.

Yolk formation is a dynamic process involving continuous changes in nutrient deposition and metabolic activity throughout the laying cycle (X. T. Liu et al., 2018). Changes in hepatic lipid synthesis, lipoprotein transport, and follicular uptake during laying progression can alter yolk lipid deposition and, consequently, the availability of lipid substrates potentially involved in volatile formation. Recent multi-omics studies have advanced the characterization of flavor-related molecular differences in poultry eggs; however, most have focused on comparisons among breeds, egg types, or production conditions at a single sampling stage (Yang et al., 2023; Zhao, 2023). Whether population-associated differences in yolk volatile profiles remain consistent or change with laying progression, and how these differences are related to lipid precursor allocation and metabolic remodeling, remain poorly understood. In particular, the relationships among potential lipid precursor pools, metabolic changes, and odor-relevant volatile compounds across laying stages have not been systematically characterized. Therefore, in this study, volatilomics, lipidomics, and widely targeted metabolomics were integrated to characterize yolk volatile profiles and associated metabolic features in four chicken populations at two sampling stages. By jointly evaluating population- and stage-associated variation, this study aimed to identify lipid and metabolic features associated with odor-relevant volatile compounds and to determine how these associations vary during laying progression.

## 2 Materials and methods

### 2.1 Chemicals and reagents

Volatile profiling was carried out using a gas chromatography–tandem mass spectrometry platform (8890 GC coupled with a 7000E triple quadrupole MS system, Agilent Technologies, Santa Clara, CA, USA) equipped with a DB-5MS fused-silica capillary column (30 m × 0.25 mm × 0.25 μm). Headspace solid-phase microextraction (HS-SPME) was performed using a 120 μm DVB/CWR/PDMS extraction fiber in combination with an automated SPME sampling system and temperature-controlled agitation module.

Lipidomics and widely targeted metabolomics analyses were performed on an ExionLC AD ultra-performance liquid chromatography system coupled to a QTRAP 6500+ tandem mass spectrometer (SCIEX, Framingham, MA, USA). Sample preparation was assisted by a high-speed refrigerated centrifuge, nitrogen-assisted vacuum concentrator, multi-position vortex mixer, ultrasonic extraction apparatus, cryogenic grinder, and precision analytical balance routinely used for metabolite extraction and processing.

### 2.2 Sample collection

A total of 48 fresh eggs were collected from four chicken populations: including Jianghan chickens (native chickens), recessive white-feathered broiler breeders (commercial broiler breeders), Lohmann Brown laying hens (commercial high-producing laying hens), and special-layer hens (Chuqin No. 2 laying hens). Each population consisted of six independent replicates, with 10 hens per replicate. Eggs were collected at 180 and 300 days of age, corresponding to the onset-of-lay and peak-laying stages, respectively, based on the laying performance of each population (Table S1). At each stage, one egg was randomly collected from each replicate, resulting in six biological replicates per population per stage (*n* = 6). The experimental replicate was considered the experimental unit. The same replicates were sampled at both stages, but individual hens within each replicate were not tracked; therefore, eggs collected at 180 and 300 days were not treated as paired observations from the same hen.

All birds were reared at the Jingshui Experimental Poultry Farm (Wuhan, China) under the same housing and management conditions, with free access to feed and water. At both sampling stages (180 and 300 d), all four chicken populations received the same laying diet, and its ingredient composition and calculated nutrient levels are provided in Table S2. Freshly collected eggs were transported to the laboratory at 4 °C, and eggs with normal external appearance and no visible defects were selected for analysis. Egg yolks were separated from the albumen, immediately freeze-dried under vacuum, ground into a fine powder, and stored at −80 °C until volatilomic, lipidomic, and widely targeted metabolomic analyses.

### 2.3 GC–MS analysis of volatile compounds

Volatile compounds in egg yolk samples were analyzed using headspace solid-phase microextraction coupled with gas chromatography–mass spectrometry (HS-SPME-GC–MS). Freeze-dried yolks were ground in liquid nitrogen to ensure homogeneity. Approximately 0.20 g of powder was placed in a 20 mL headspace vial, followed by addition of 0.20 g NaCl to promote volatile partitioning. Samples were incubated at 60 °C for 5 min for equilibration. Volatiles were then extracted using a DVB/CWR/PDMS fiber (120 μm; Agilent Technologies) exposed to the headspace for 15 min at 60 °C. After extraction, the fiber was thermally desorbed in the GC inlet at 250 °C for 5 min under splitless mode.

GC–MS analysis was performed using an Agilent 8890 GC system coupled to a 7000 series triple-quadrupole mass spectrometer equipped with a DB-5MS capillary column (30 m × 0.25 mm × 0.25 μm). Helium (99.999%) was used as the carrier gas at 1.2 mL/min. The oven program was set as follows: 40 °C (3.5 min), ramped to 100 °C at 10 °C/min, to 180 °C at 7 °C/min, and to 280 °C at 25 °C/min with a final hold of 5 min. The injector, ion source, quadrupole, and transfer line temperatures were 250 °C, 230 °C, 150 °C, and 280 °C, respectively. Mass spectrometric detection was performed in electron ionization mode at 70 eV using selected ion monitoring (SIM).

Volatile compounds were tentatively annotated using a curated in-house database established on the basis of mass spectral information, retention indices (RIs), literature data, and available reference information (Yuan et al., 2024). During database construction, mass spectra were compared with the NIST 2020 mass spectral library using a minimum matching factor of 70. RIs were calculated using a C7–C40 *n*-alkane series analyzed under the same chromatographic conditions and compared with reference RI values. During sample analysis, target compounds were monitored in selected ion monitoring (SIM) mode within predefined retention windows using compound-specific quantitative and qualitative ions. Compound annotation was based on agreement of the observed retention time with the expected retention time in the in-house database and detection of the predefined characteristic ions in the background-subtracted mass spectrum. As authentic standards were not independently analyzed alongside the samples in the present study, all detected volatile compounds were considered tentatively annotated. Compound-specific annotation information, including retention time, calculated and reference RIs, and quantitative and qualitative ions, is provided in Table S3.

Semi-quantitative analysis was performed using benzaldehyde-d₆ as a single internal standard. The estimated concentration of each volatile compound was calculated from the peak-area ratio of the analyte to the internal standard according to the following equation:

$$X_{i}=\frac{V_{s}\times C_{s}}{M}\times \frac{I_{i}}{I_{s}}$$

where $X_{i}$ represents the estimated concentration of compound i(μg/g), $V_{s}\;$and $C_{s}\;$represent the volume and concentration of the internal standard solution, respectively; Mis the sample mass, and $I_{i}$ and $I_{s}\;$represent the peak areas of the target analyte and internal standard, respectively. Because a single internal standard was used, the calculated values were regarded as semi-quantitative estimates rather than absolute concentrations, as individual volatile compounds may differ in SPME extraction efficiency and MS response.

### 2.4 Odor activity value (OAV) determination

To estimate the potential sensory contribution of individual volatile compounds to egg yolk aroma, odor activity values (OAVs) were calculated by dividing the semi-quantitatively estimated concentration of each compound by its corresponding odor threshold reported in the literature:

$$\mathrm{OA}V_{i}=\frac{C_{i}}{T_{i}}$$

where $C_{i}\;$represents the semi-quantitatively estimated concentration of volatile compound i(μg/g), and $T_{i}$ represents its odor threshold value in water obtained from the literature. Compounds with calculated OAVs ≥1 were considered to have potential contributions to egg yolk aroma and were prioritized for subsequent analysis.

### 2.5 Lipidomics analysis

Freeze-dried yolk samples were thawed at 4 °C and ∼ 20 mg of powder was extracted with methyl tert-butyl ether/methanol (3:1, *v*/v) containing internal standards. After vortexing, water was added for phase separation, and samples were centrifuged at 4000 ×*g* for 10 min. The upper organic phase was collected, dried under nitrogen, and reconstituted in acetonitrile/isopropanol (1:1, *v*/v) for LC–MS/MS analysis. Lipid profiling was performed using an ExionLC AD system coupled with a QTRAP 6500+ mass spectrometer (SCIEX, USA). Separation was achieved on an Accucore C30 column (2.1 × 100 mm, 2.6 μm; Thermo Fisher Scientific) using acetonitrile/water (60:40, *v*/v, A) and acetonitrile/isopropanol (10:90, *v*/v, B), both containing 0.1% formic acid and 10 mM ammonium formate. The flow rate was 0.35 mL/min, column temperature 45 °C, and injection volume 2 μL. Data were acquired in both positive and negative electrospray ionization modes using multiple reaction monitoring (MRM). Source temperature was 500 °C, with spray voltages of +5500 V and − 4500 V. Lipids were detected and annotated using a curated in-house database (MWDB, MetWare) based on predefined precursor ion (Q1)–product ion (Q3) transitions and retention times (RTs), with characteristic lipid-fragmentation patterns and reference-standard information incorporated into the database where available. Relative lipid abundances were determined by internal-standard normalization.

### 2.6 Widely targeted metabolomics analysis

Approximately 20 mg of freeze-dried yolk powder was extracted with 400 μL of pre-cooled methanol/water (7:3, *v*/v) containing internal standards. Samples were vortexed, ultrasonicated in an ice-water bath, and incubated at −20 °C to precipitate proteins. After centrifugation at 12,000 rpm (4 °C), the supernatant was collected and further clarified by a second centrifugation prior to LC–MS/MS analysis. Metabolomic profiling was performed using an ExionLC AD UPLC system coupled with a QTRAP LC–MS/MS platform (SCIEX, USA). Separation was carried out on a Waters ACQUITY HSS T3 C18 column (2.1 × 100 mm, 1.8 μm). The mobile phases consisted of water with 0.1% formic acid (A) and acetonitrile (B), with a gradient from 5% to 99% B. The flow rate was 0.4 mL/min, column temperature 40 °C, and injection volume 2 μL. Data were acquired in both positive and negative electrospray ionization modes using multiple reaction monitoring (MRM). The ion source temperature was 500 °C, with spray voltages set at +5500 V and − 4500 V. Declustering potentials and collision energies were optimized for each transition.

Metabolites were identified based on retention time (RT), precursor/product ion pairs, MS/MS spectral information, and an in-house metabolite database (MWDB, Metware Biotechnology Co., Ltd., Wuhan, China), with reference MS/MS spectral information incorporated into the database-supported annotation procedure. Relative metabolite abundances were obtained from integrated MRM peak areas. Quality-control (QC) samples were prepared by pooling equal volumes of extracts from randomly selected study samples and were inserted approximately once every 10 analytical samples to monitor analytical reproducibility and instrument stability. Missing values in the raw dataset were imputed using the k-nearest neighbors (KNN) algorithm. The coefficient of variation (CV) of each metabolite was subsequently calculated across QC samples, and metabolites with QC CV ≥ 30% were excluded. After QC-based filtering, 926 metabolites were retained for subsequent analyses. No additional normalization or analytical drift correction was applied.

### 2.7 Statistical analysis

All data were expressed as mean ± standard error (SEM). The experimental replicate was considered the experimental unit, with one egg from each replicate used for omics analyses (*n* = 6 per chicken population per laying stage). To account for the factorial design and repeated sampling of the same experimental replicates across stages, linear mixed-effects models were used to evaluate the effects of chicken population, sampling stage, and their interaction. Population, stage, and population × stage were included as fixed effects, whereas replicate nested within population was included as a random effect. When significant main effects or population × stage interactions were detected, post hoc or simple-effect comparisons were performed using estimated marginal means, as appropriate. Comparisons among chicken populations within each sampling stage were adjusted using Tukey's method, whereas comparisons between sampling stages within each chicken population were adjusted using the Holm method. For each omics dataset, feature-wise *P* values obtained from the factorial models for population, sampling stage, and population × stage effects were separately adjusted using the Benjamini–Hochberg false discovery rate (FDR) procedure. Differential features identified in the multivariate analyses were re-evaluated using the revised model-based statistics, and the factorial and FDR-adjusted results were used for statistical interpretation of population, stage, and interaction effects. The complete factorial results are provided in Tables S4-S6.

Principal component analysis (PCA) was used to assess overall variation in the datasets, whereas partial least squares discriminant analysis (PLS-DA) was used to visualize supervised multivariate patterns. PCA and PLS-DA were performed using the MetaboAnalyst platform. PLS-DA models were evaluated using seven-fold cross-validation, with model performance assessed by R^2^X, R^2^Y, and Q^2^ values, and were further assessed by 200-permutation tests for potential overfitting (Tables S7, and Fig. S1). Pearson correlation analysis was used to evaluate associations among volatile compounds, lipids, and metabolites. Statistical analyses, correlation matrices, and network visualizations were performed using R software (version 4.4.3).

## 3 Results and discussion

### 3.1 Profiling of volatile molecular features in egg yolks across chicken populations and laying stages

Headspace solid-phase microextraction coupled with gas chromatography–mass spectrometry (HS-SPME-GC–MS) was applied to characterize the volatile profiles of egg yolks from four chicken populations at the onset-of-lay (180 d) and peak-laying (300 d) stages. A representative total ion chromatogram (TIC) obtained from the HS-SPME-GC–MS analysis, together with reference mass spectra used to support the annotation of four representative odor-relevant aldehydes (2-undecenal, heptanal, nonanal, and (E)-2-octenal), is provided in Fig. S2. Using the SIM-based GC–MS workflow, 265 volatile compounds were tentatively annotated and classified into several chemical classes, including hydrocarbons, aldehydes, alcohols, ketones, terpenoids, esters, heterocyclic compounds, acids, sulfur-containing compounds, and halogenated compounds (Fig. 1A). Hydrocarbons represented the largest chemical class, followed by terpenoids, aldehydes, and alcohols. Among these, aldehydes and alcohols derived from unsaturated fatty acid oxidation are recognized as important contributors to egg aroma because of their low odor thresholds (Chang et al., 2026; Li et al., 2025). The relative distribution of these volatile classes differed among chicken populations and between sampling stages, indicating variation in yolk volatile composition. These differences were further examined at the individual-compound level to characterize population- and stage-associated patterns.

**Fig. 1:**
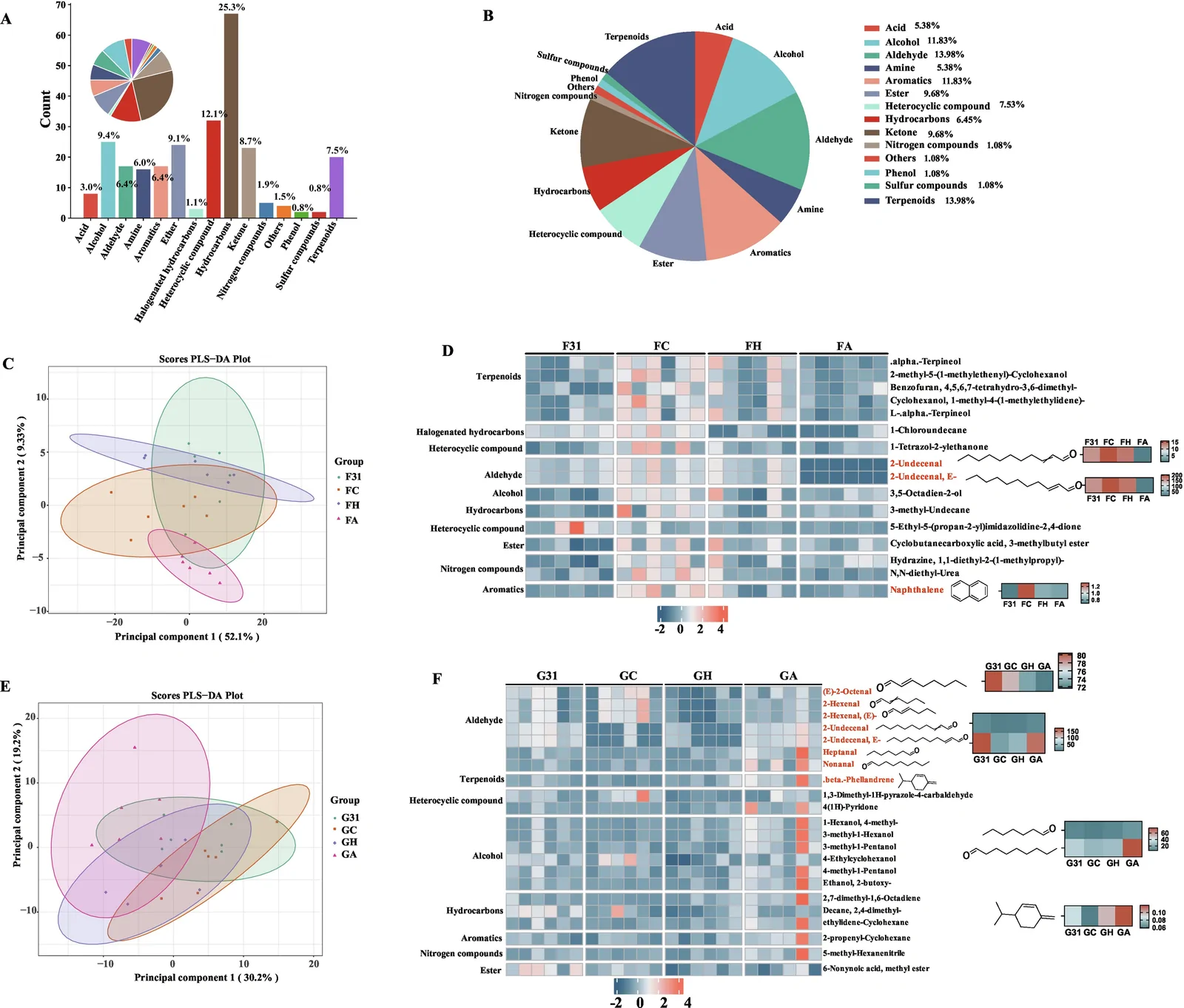
**Volatile profiling of egg yolks from different chicken populations at the onset-of-lay and peak-laying stages determined by HS-SPM*E*-GC–MS.** (A) Classification and relative abundance of volatile compounds tentatively annotated in egg yolks. (B) Distribution of volatile compound classes across different chicken populations and laying stages. (C) PLS-DA score plots of volatile profiles among onset-of-lay egg yolks, including Chuqin No. 2 (F31), white broilers (FC), high-yield layers (FH), and local chickens (FA). (D) Heatmap of differential volatile compounds among onset-of-lay groups. (E) PLS-DA score plots of volatile profiles among peak-laying egg yolks, including Chuqin No. 2 (G31), white broilers (GC), high-yield layers (GH), and local chickens (GA). (F) Heatmap of differential volatile compounds among peak-laying groups.

To examine population-associated variation, partial least squares discriminant analysis (PLS-DA) was performed separately at the two sampling stages. At the onset-of-lay stage, the PLS-DA score plot revealed differences in overall volatile profiles among native chickens (FA), commercial broiler breeders (FC), and commercial high-producing laying hens (FH), whereas Chuqin No. 2 (F31) occupied an intermediate position with partial overlap among the other populations (Fig. 1C). Heatmap analysis further showed population-associated variation in individual volatile compounds (Fig. 1D). Compared with FA, FC exhibited higher abundances of several terpenoid compounds and lipid oxidation-related aldehydes, whereas F31 displayed an intermediate volatile profile characterized by increased abundance of 2-undecenal relative to FA but lower levels of terpenoid compounds than FC. Together, these results indicate that population-associated differences in yolk volatile composition were already evident at the onset-of-lay stage. Previous studies have linked genetic differences among chicken populations to variation in lipid metabolism, fatty acid composition, and yolk lipid deposition (X.-t. Liu et al., 2018; Zhang et al., 2026). Such variation may contribute to differences in the abundance and distribution of potential volatile precursor pools. In the present study, this possibility was further examined by integrating the volatile profiles with lipidomic and metabolomic features, rather than assuming a direct genetic or precursor–product relationship from the volatile data alone.

Population-associated variation in volatile composition remained evident during peak laying. The PLS-DA score plot revealed differences in overall volatile profiles among the peak-laying groups, with native chickens (GA) and commercial broiler breeders (GC) showing the greatest difference, whereas Chuqin No. 2 (G31) and commercial high-producing laying hens (GH) occupied intermediate positions (Fig. 1E). Heatmap analysis further showed population-associated variation in individual volatile compounds (Fig. 1F). GA contained higher abundances of several lipid oxidation-related aldehydes, including heptanal and nonanal, together with several fatty alcohols associated with green and fatty aroma notes. Aldehydes such as heptanal and nonanal can arise from the oxidative degradation of unsaturated lipid substrates (Chen et al., 2023), suggesting a potential association between yolk lipid composition and the observed volatile profiles. Similar variation in lipid-derived aldehydes has also been reported among poultry populations and has been associated with differences in egg flavor characteristics (Zhao et al., 2025). Overall, egg yolk volatile composition varied among chicken populations and between laying stages. Population-associated differences were evident at both sampling stages, while several lipid oxidation-related volatiles showed stage-associated changes. These observations motivated subsequent analyses of compounds with calculated OAV ≥ 1 and of the lipid and metabolic features associated with volatile variation.

### 3.2 Characterization of volatile compounds with potential aroma contributions

#### 3.2.1 Characterization of volatile compounds

To evaluate the potential contributions of volatile compounds to egg yolk aroma, OAVs were calculated using the semi-quantitatively estimated concentrations and odor thresholds reported in the literature. Among the 265 detected volatile compounds, literature odor thresholds were available for 93 compounds, allowing their OAVs to be calculated (Fig. 1B). These compounds comprised aldehydes, terpenoids, alcohols, aromatics, ketones, and esters. Aldehydes and terpenoids were the predominant classes (13.98% each), followed by alcohols and aromatics (11.83% each), and ketones and esters (9.68% each), indicating that the compounds for which OAVs could be calculated were distributed across multiple chemical classes. Among the compounds with calculated OAVs ≥1, aldehydes were prominent, particularly heptanal, nonanal, (E)-2-octenal, and 2-undecenal isomers. These aldehydes can arise from the oxidative degradation of unsaturated lipid substrates, including linoleic acid- and oleic acid-containing lipids, and are associated with fatty, green, citrus-like, and roasted aroma notes (Zhang, Liu, Yang, Huang, Liu, Huang, Sun, & Li, 2018). Terpenoids are generally associated with floral and fruity notes, whereas alcohols and aromatic compounds may further contribute to aroma complexity. Notably, several lipid oxidation-derived aldehydes, including heptanal, nonanal, and (E)-2-octenal, consistently had calculated OAVs ≥1 across chicken populations and sampling stages. Their abundance patterns may be associated with variation in oxidation-sensitive lipid pools among populations and stages (Yang et al., 2023). These observations provided a basis for subsequent lipidomic analyses of lipid features associated with volatile variation.

#### 3.2.2 Population- and stage-associated variation in volatile profiles

Factorial mixed-effects analysis identified 3, 50, and 9 volatile compounds showing significant population, sampling-stage, and population × stage effects, respectively, after FDR correction (Table S4). These results indicated that sampling stage was associated with a broader range of VOC changes, whereas a smaller subset of compounds exhibited population-dependent responses across stages. PLS-DA and heatmap analyses were further used to characterize population-associated differences in volatile profiles at each sampling stage. At the onset-of-lay stage, PLS-DA visualized population-associated differences in the multivariate volatile profiles (Fig. 2C, E, G), with commercial broiler breeders (FC) and commercial high-producing laying hens (FH) clearly separated from native chickens (FA), whereas Chuqin No. 2 (F31) occupied an intermediate position, indicating differences in odor-relevant volatile composition among chicken populations (Yang et al., 2023). Heatmap analysis further revealed population-associated accumulation patterns of odor-relevant compounds (Fig. 2D, F, H). Compared with FA, FC, FH, and F31 exhibited higher abundances of 2-undecenal and (E)-2-undecenal, both of which are recognized as important lipid oxidation-derived aldehydes contributing to fatty and green aroma notes (Zhou et al., 2024). The calculated OAVs of (E)-2-undecenal reached 203.12 and 177.52 in FC and FH, respectively, compared with 2.83 in FA, indicating substantial differences in its potential contribution to aroma among chicken populations. These findings indicate that population-associated differences in odor-relevant aldehydes were already detectable at the onset of lay, with 2-undecenal isomers showing particularly distinct abundance patterns among chicken populations.

**Fig. 2:**
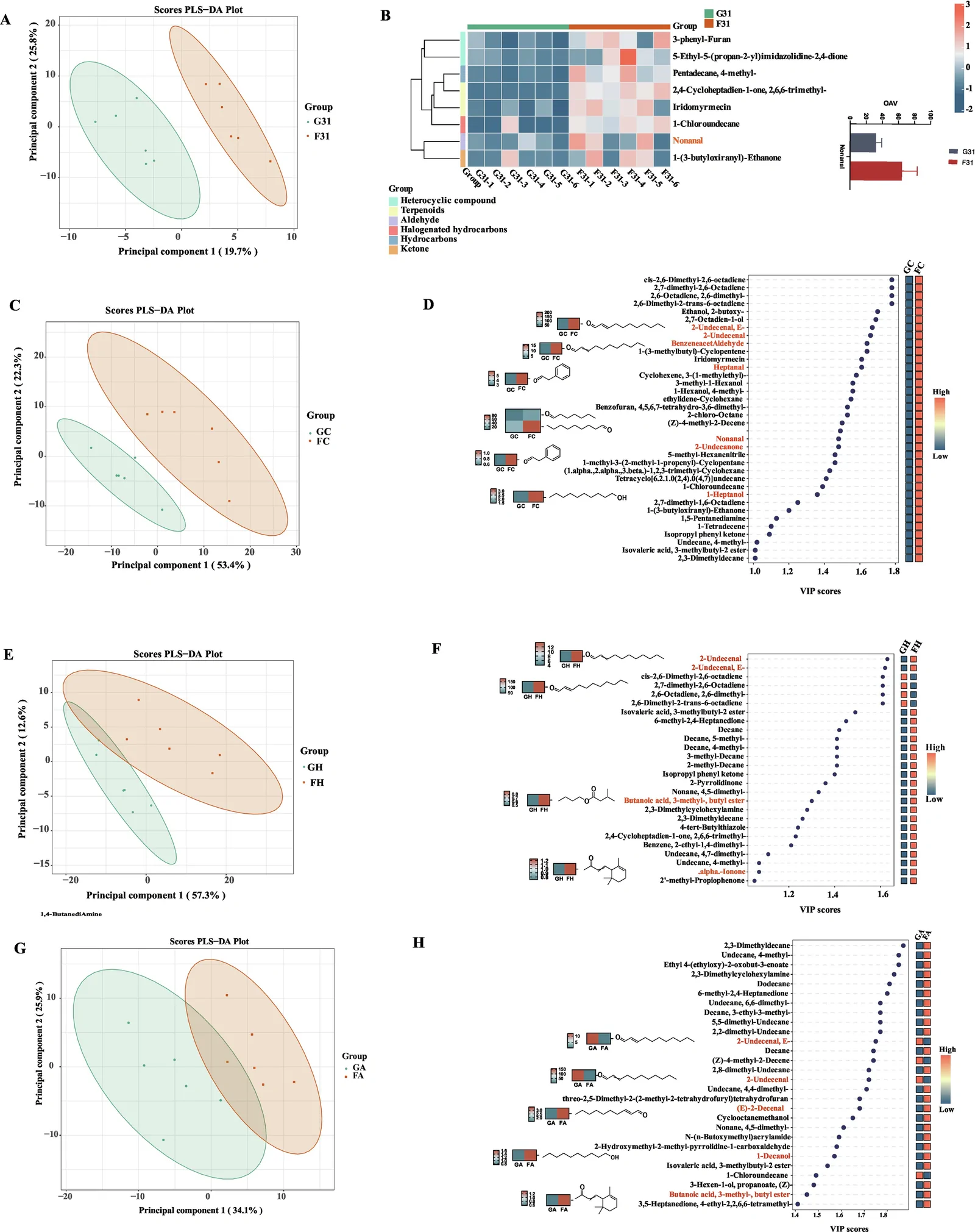
**Population- and stage-associated variation in odor-relevant volatile compounds of egg yolks determined by GC–MS and odor activity value (OAV) analysis.** (A) PLS-DA score plots comparing Chuqin No. 2 egg yolks collected at the onset-of-lay (F31) and peak-laying (G31) stages. (B) Heatmap and OAVs of differential odor-relevant volatile compounds between F31 and G31. (C) PLS-DA score plots of odor-relevant volatile profiles among peak-laying groups (G31, GC, GH, and GA). (D) VIP scores and heatmap of differential odor-relevant volatile compounds among peak-laying groups. (E) PLS-DA score plots comparing high-yield layers (FH) and local chickens (FA) at the onset-of-lay stage. (F) VIP scores and heatmap of differential odor-relevant volatile compounds between FH and FA. (G) PLS-DA score plots comparing local chicken egg yolks at the onset-of-lay (FA) and peak-laying (GA) stages. (H) Heatmap of differential odor-relevant volatile compounds between FA and GA.

At peak laying, population-associated differences were also observed in the profiles of several odor-relevant aldehydes. PLS-DA revealed clearer separation between native chickens (GA) and the commercial populations (GC and GH), whereas Chuqin No. 2 (G31) exhibited a volatile profile more similar to the commercial groups (Fig. 2C, E). Heatmap and VIP analyses highlighted heptanal, nonanal, (E)-2-octenal, and (E)-2-hexenal as representative discriminant aldehydes (Fig. 2D, F, H). These aldehydes generally showed higher abundances in GC, GH, and G31 than in GA, indicating population-associated differences in lipid oxidation-derived volatile profiles at this sampling stage. Among them, (E)-2-hexenal showed higher calculated OAVs in the commercial populations and Chuqin No. 2, suggesting a greater potential contribution to green and fatty aroma characteristics during peak laying. Because these aldehydes are commonly associated with the oxidation of unsaturated fatty acids, their differential abundance may be associated with differences in oxidation-sensitive lipid pools among chicken populations (Chen et al., 2023). Overall, volatile profiles exhibited both population- and stage-associated variation, with factorial analysis indicating a broader effect of sampling stage and significant population × stage interactions for a subset of VOCs. Population-associated differences in several odor-relevant aldehydes were detectable at both sampling stages, while changes in lipid oxidation-derived volatiles accompanied laying progression. These patterns suggest that changes in potential lipid-derived volatile precursor pools accompany laying progression, providing a basis for subsequent investigation of lipid remodeling associated with volatile variation.

#### 3.2.3 Stage-associated changes in odor-relevant volatile compounds

Laying progression was associated with marked changes in egg yolk volatile profiles. PLS-DA revealed separation between onset-of-lay (F groups) and peak-laying (G groups) samples across all chicken populations, indicating stage-associated changes in volatile composition (Fig. 2A). Factorial analysis showed that sampling stage was associated with a broader range of significant volatile changes than population or population × stage effects (Table S4). Heatmap analysis further showed differences in the abundance patterns of multiple odor-relevant compounds between the two sampling stages, including several aldehydes and terpenoid-related volatiles (Fig. 2B), indicating stage-associated changes in the abundance of compounds with potential aroma contributions.

Among the representative compounds, several lipid oxidation-derived aldehydes, including heptanal, nonanal, and (E)-2-hexenal, showed higher abundances at peak laying than at onset of lay in the corresponding comparisons. These aldehydes are commonly associated with the oxidation of unsaturated fatty acids, and contribute fatty, green, and fresh aroma notes to egg yolks (Chen et al., 2023). Notably, the calculated OAV of (E)-2-hexenal in Chuqin No. 2 increased from 166.44 at the onset of lay to 947.74 during peak laying, suggesting an increased potential contribution to aroma at the later sampling stage. Factorial analysis further supported a significant overall stage effect for (E)-2-hexenal after FDR correction (Table S4). Heptanal and nonanal also showed increasing abundance patterns in several chicken populations, although their overall stage effects did not remain significant after FDR correction (Yang et al., 2019). Overall, the transition between the two sampling stages was accompanied by broad changes in volatile composition, including changes in several lipid oxidation-derived aldehydes. These observations indicate that sampling stage is associated with changes in odor-relevant volatiles and provide a basis for subsequent analyses of lipid remodeling and metabolic changes associated with aroma variation.

### 3.3 Population- and stage-associated remodeling of the egg yolk lipidome

#### 3.3.1 Population-associated lipid variation at the onset of lay

To investigate the metabolic basis underlying population-associated differences in egg yolk aroma, lipidomic profiling was performed to characterize yolk lipid composition at the onset-of-lay stage. Representative total ion chromatograms (TICs) and MRM detection profiles obtained in negative- and positive-ion modes during the LC–MS/MS lipidomic analysis are provided in Fig. S3. A total of 1268 lipid species were identified (Fig. 3A), with glycerophospholipids accounting for the largest proportion (74.67%), followed by sphingolipids (13.41%) and fatty acyls (6.81%). Among the glycerophospholipids, phosphatidylcholines (PCs) and phosphatidylethanolamines (PEs) were the predominant subclasses, consistent with their essential roles in membrane organization and lipid transport during yolk formation (Luo et al., 2023). Because several odor-relevant aldehydes identified in this study, including heptanal, nonanal, and (E)-2-octenal, originate from oxidation of PUFA-containing lipids (Zhang, Liu, Yang, Huang, Liu, Huang, Sun, Li, & chemistry, 2018), variation in lipid composition is expected to influence the availability of volatile precursors. PLS-DA revealed clear separation of lipidomic profiles among chicken populations at the onset of lay (Fig. S4A). Native chickens (FA) were distinctly separated from the commercial populations (FC and FH), whereas Chuqin No. 2 (F31) exhibited an intermediate lipidomic profile, indicating population-associated differences in yolk lipid composition during early laying.

**Fig. 3:**
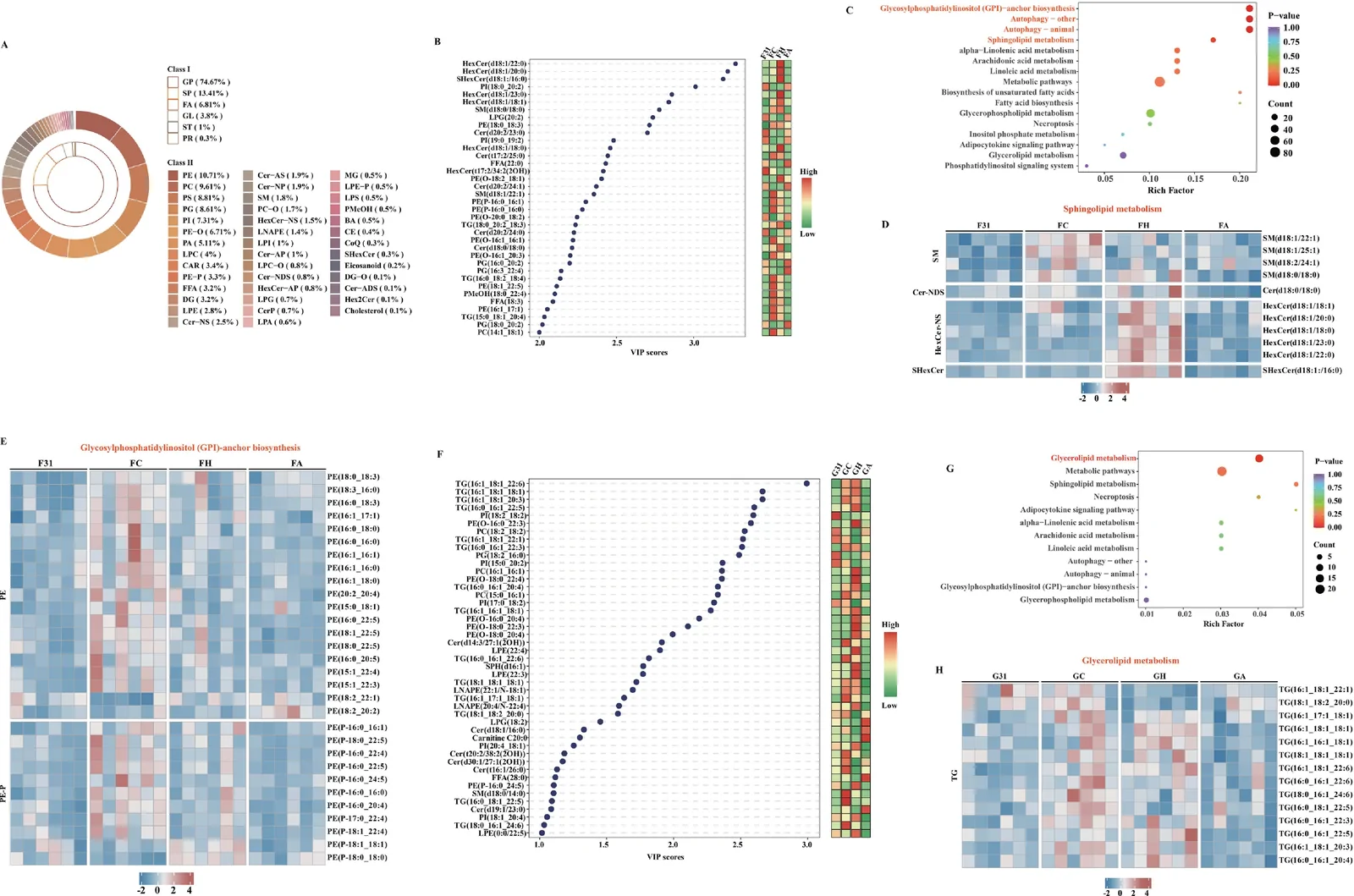
**Population- and stage-associated variation in egg yolks revealed by lipidomic analysis.** (A) Classification and relative abundance of lipid species identified in egg yolks. (B) VIP scores and heatmap of differential lipid species among onset-of-lay groups (F31, FC, FH, and FA). (C) KEGG pathway enrichment analysis of differential lipid species in onset-of-lay groups. (D) Heatmap of sphingolipid species, including sphingomyelins (SM), ceramides (Cer), hexosylceramides (HexCer), and sulfatides (SHexCer), among onset-of-lay groups. (E) Heatmap of phosphatidylethanolamine (PE) and plasmalogen phosphatidylethanolamine (PE-P) species among onset-of-lay groups. (F) VIP scores and heatmap of differential lipid species among peak-laying groups (G31, GC, GH, and GA). (G) KEGG pathway enrichment analysis of differential lipid species in peak-laying groups. (H) Heatmap of triacylglycerol (TG) species among peak-laying groups.

Using the original multivariate screening criteria (VIP > 1 and nominal *P* < 0.05), 138 lipid species were identified as discriminant features among populations at the onset-of-lay (Fig. 3B). These multivariate patterns were further evaluated using model-based simple-effect comparisons with FDR correction (Table S5). KEGG enrichment analysis indicated that these lipids were mainly associated with sphingolipid metabolism, glycosylphosphatidylinositol (GPI)-anchor biosynthesis, and α-linolenic acid metabolism (Fig. 3C), suggesting extensive remodeling of membrane-associated lipid pathways during early yolk development (Cartoni Mancinelli et al., 2022). Heatmap analysis further revealed population-associated abundance patterns of several glycerophospholipid and sphingolipid subclasses, including PCs, ether-linked PCs (PC-Os), PEs, PE plasmalogens (PE-Ps), and sphingomyelins (SMs) (Fig. 3D, E and Fig. S4C, D). These patterns were broadly consistent with population-associated variation in several odor-relevant aldehydes observed in the volatilomic analysis, suggesting differences in the availability of oxidation-sensitive lipid substrates among chicken populations. Glycerophospholipids constitute the major structural lipids of biological membranes and are important components of lipoproteins responsible for lipid transport during yolk formation (Hannun & Obeid, 2018). PUFA-containing PE species and plasmalogens are particularly susceptible to oxidative degradation and therefore represent important precursors for lipid oxidation-derived volatile compounds (Kong et al., 2025; Zhao et al., 2025). The enrichment of these lipid subclasses in FC, FH, and F31 was consistent with the higher abundance of odor-relevant aldehydes observed in the volatilomic analysis, suggesting differences in the abundance of oxidation-sensitive lipid pools among these populations. Collectively, these results indicate that population-associated lipidomic differences at the onset of lay were particularly evident in membrane-associated lipid pools. Such variation may contribute to differences in the availability of oxidation-sensitive lipid substrates for volatile formation.

#### 3.3.2 Population-associated lipid variation is reduced at the peak laying

At the peak laying, population-associated lipidomic differences were less extensive than those observed at the onset of lay. Principal component analysis (PCA) showed relatively closer clustering of several populations (Fig. S4B), consistent with the reduced number of lipids showing significant population differences in model-based simple-effect comparisons after FDR correction (Table S5). Using the original multivariate screening criteria (VIP > 1 and nominal *P* < 0.05), 45 discriminant lipid species were identified among the 300-day groups (Fig. 3F), compared with 138 at 180 days. KEGG enrichment analysis highlighted glycerolipid metabolism among the pathways associated with lipid variation at this stage (Fig. 3G), suggesting a greater contribution of neutral lipid metabolism at this stage (Fu et al., 2022).

Heatmap analysis further revealed population-associated patterns of triacylglycerol (TG) abundance (Fig. 3H). GC, GH, and G31 generally showed higher abundances of several long-chain and unsaturated TG species than GA. As the principal storage form of fatty acids in egg yolk, TGs constitute an important reservoir of unsaturated fatty acids and may contribute to oxidation-sensitive lipid pools. The abundance patterns of these TG species were broadly consistent with those of several lipid oxidation-derived aldehydes observed in the volatilomic analysis, suggesting an association between neutral lipid composition and potential volatile precursor availability (Zhang, Liu, Yang, Huang, Liu, Huang, Sun, & Li, 2018). In particular, linoleic acid- and oleic acid-containing TG species are potential sources of odor-relevant aldehydes, including heptanal, nonanal, and (E)-2-octenal (Yang et al., 2019). The higher abundance of these TG species in GC, GH, and G31 was consistent with the greater accumulation of lipid oxidation-derived aldehydes observed in the volatilomic analysis, supporting a close association between neutral lipid deposition and aroma precursor availability. Collectively, these findings indicate that population-associated lipidomic variation was less extensive at the peak laying than at the onset of lay, while glycerolipid metabolism and TG-associated differences remained prominent features of the later-stage lipidome.

#### 3.3.3 Stage is associated with extensive remodeling of the yolk lipidome

Factorial mixed-effects analysis identified 644 lipid species with significant sampling-stage effects after FDR correction (Table S5), indicating that sampling stage was associated with the largest number of significant lipid changes. To further characterize these changes, lipidomic profiles were compared between the onset-of-lay and peak-laying stages within each chicken population (Fig. 4A-D). PCA revealed clear stage-associated shifts in yolk lipidomic profiles. Model-based simple-effect analysis further identified 112, 249, 117, and 204 lipid species showing significant stage-associated differences after FDR correction in populations A, C, H, and 31, respectively (Table S5). Stage-associated lipidomic changes were evident across all four chicken populations, although the number and identity of significantly altered lipid species varied among populations (Chen et al., 2024). Although the specific sets of stage-associated lipids differed among populations, no individual lipid retained a significant population × stage interaction after FDR correction, indicating that the dominant lipidomic shift was associated with sampling stage rather than a robust population-specific stage response.

**Fig. 4:**
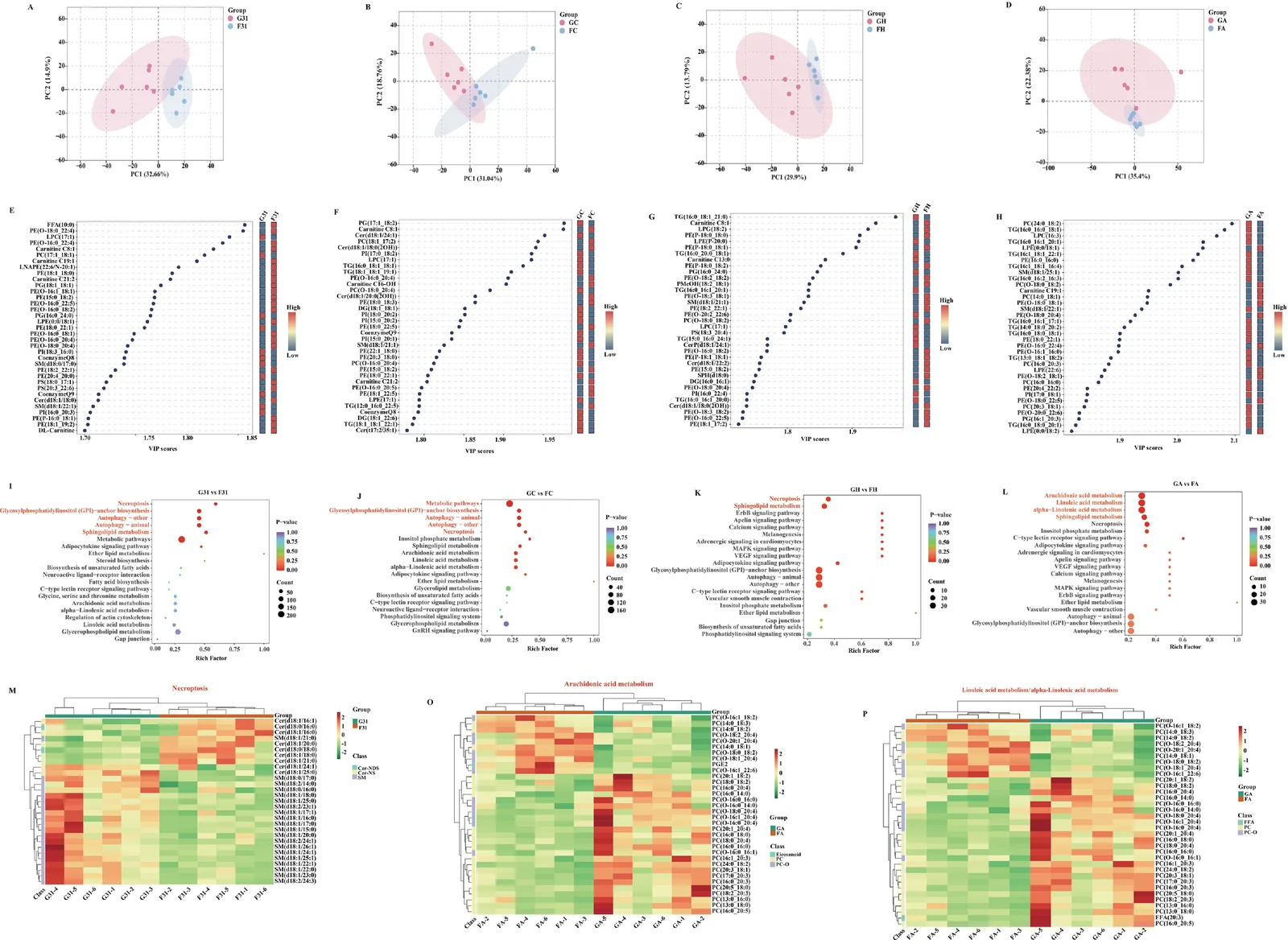
**Stage-associated lipid remodeling in egg yolks across different chicken populations revealed by lipidomic analysis.** (A-D) PLS-DA score plots comparing lipidomic profiles between the onset-of-lay and peak-laying stages of Chuqin No. 2 (F31 vs. G31), white broilers (FC vs. GC), high-yield layers (FH vs. GH), and local chickens (FA vs. GA), respectively. (E-H) VIP scores and heatmaps of differential lipid species identified in each stage comparison. (I-L) KEGG pathway enrichment analysis of differential lipid species. (M-P) Heatmaps of representative lipid species involved in significantly enriched metabolic pathways.

VIP analysis and hierarchical clustering identified extensive alterations in membrane-associated lipid classes, including phosphatidylethanolamines (PEs), phosphatidylcholines (PCs), sphingomyelins (SMs), and ceramides (Cers) (Fig. 4E-H). Compared with onset-of-lay yolks, peak-laying yolks showed pronounced remodeling of these lipid species (Fig. S5), indicating extensive changes in membrane-associated lipid composition between the two sampling stages. Pathway enrichment highlighted glycerophospholipid metabolism, sphingolipid metabolism, arachidonic acid metabolism, and linoleic acid metabolism in population-specific stage comparisons (Fig. 4I-L), suggesting that the stage-associated lipidomic shift involved both membrane lipid organization and unsaturated fatty acid-containing lipid pools (Yang et al., 2025). Among these, sphingolipid metabolism was consistently enriched across all chicken populations and was accompanied by increased abundances of SM and Cer species during peak laying (Fig. 4M-P and Fig. S4E, S5C-E). As structural components of biological membranes and lipoproteins, these lipid classes are closely associated with membrane organization and lipid transport during yolk formation (Tardieu et al., 2021). In addition, increased abundances of PE-containing lipid species further support active membrane phospholipid remodeling during peak laying (Fig. S4D—F).

Membrane lipid remodeling may influence the availability of oxidation-sensitive fatty acids. PUFA-containing PE and PC species are susceptible to oxidative degradation and can provide substrates for the formation of lipid oxidation-derived aldehydes (Yu et al., 2023). Consistent with this possibility, stage-associated changes involved multiple PUFA-containing phospholipids and pathways related to glycerophospholipid and unsaturated fatty acid metabolism, particularly in commercial breeds and Chuqin No. 2 (Fig. S4E-G). These lipidomic changes occurred alongside the stage-associated variation in odor-relevant aldehydes identified by volatilomic analysis. Together, these findings indicate extensive stage-associated remodeling of membrane phospholipids and neutral lipid pools. Variation in these oxidation-sensitive lipid substrates provides a plausible metabolic link between yolk lipid composition and the stage-associated changes in volatile profiles.

### 3.4 Population- and stage-associated remodeling of the yolk metabolome

#### 3.4.1 Population-associated metabolic variation related to precursor transport and lipid remodeling

To further characterize the metabolic variation associated with population differences in egg yolk volatile profiles, widely targeted metabolomics was performed to characterize small-molecule metabolites associated with nutrient transport, lipid metabolism, and precursor allocation. Representative total ion chromatograms (TICs) and MRM detection profiles obtained in negative- and positive-ion modes during the widely targeted LC–MS/MS metabolomics analysis are provided in Fig. S6. After QC-based filtering, 926 metabolites were identified, encompassing amino acids, organic acids, carbohydrates, lipids, nucleotides, and small peptides. Factorial mixed-effects analysis identified 15, 275, and 17 metabolites showing significant population, sampling-stage, and population × stage effects, respectively, after FDR correction (Table S6). These results indicated that sampling stage was associated with a broader range of metabolic changes, whereas population-associated effects were more restricted and a subset of metabolites exhibited population-dependent stage responses. Multivariate analysis further revealed population-associated differences in metabolic profiles at both sampling stages (Fig. 5A-B). Heatmap analysis showed distinct patterns of amino acid derivatives, sugar alcohols, phospholipid-related metabolites, and carnitine-associated metabolites among chicken populations, suggesting differences in nutrient utilization and metabolic allocation. At the onset-of-lay stage, 83 discriminant metabolites were identified using the original multivariate screening criteria, and KEGG enrichment analysis highlighted ABC transporters and glycerophospholipid metabolism (Fig. 5C-D). These pathway patterns were consistent with differences in metabolite transport and phospholipid-related metabolism among populations (Liu et al., 2025). Metabolites associated with ABC transporter pathways also displayed distinct abundance patterns among chicken populations (Fig. 5E). FA contained higher levels of several dipeptides, including Phe-Leu and Cyclo(Ala-Pro), together with the sugar alcohols mannitol and sorbitol, whereas the other populations showed relatively higher abundances of several lipid-associated metabolites.

**Fig. 5:**
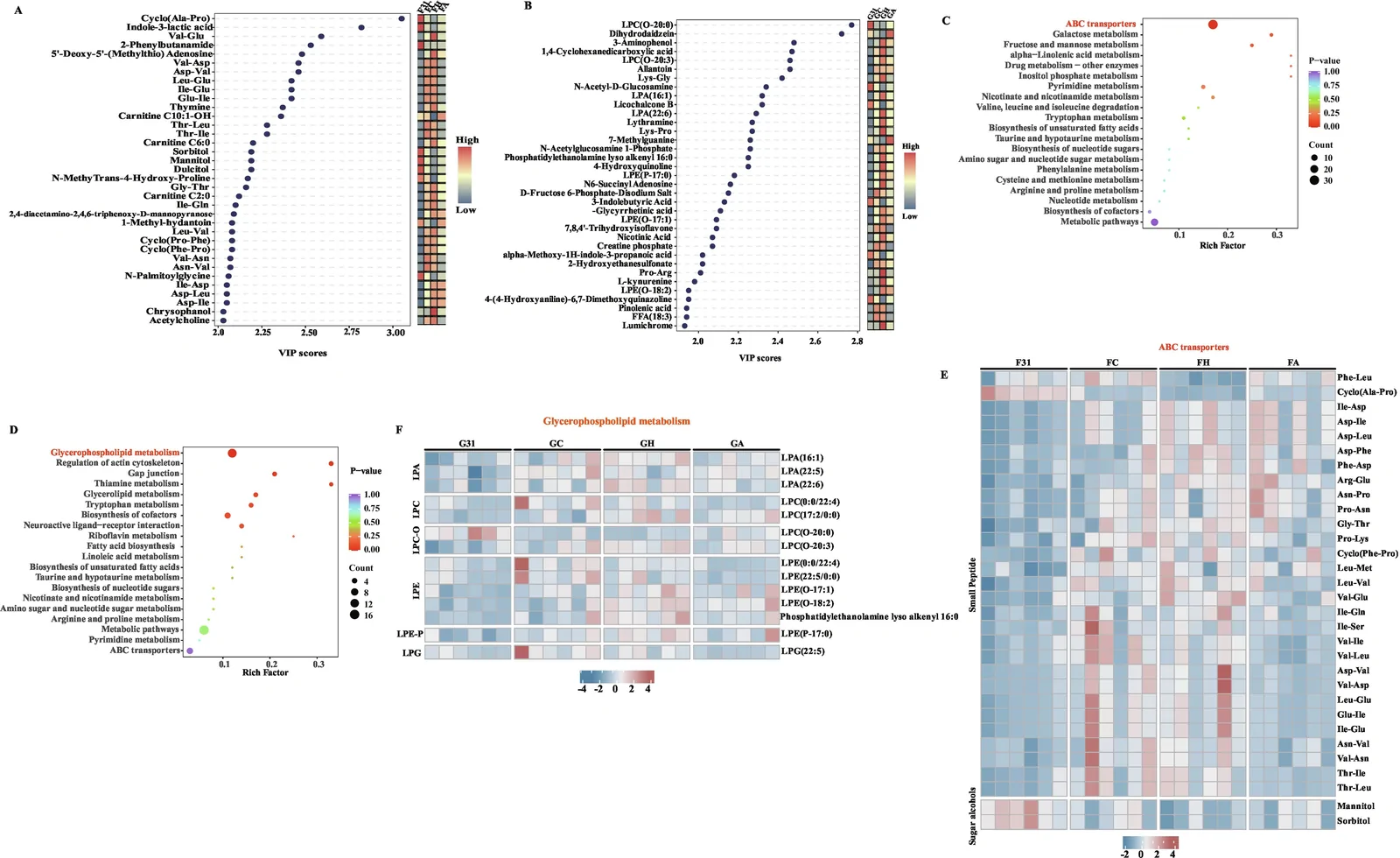
**Metabolomic characterization of population-associated variation in egg yolks at the onset-of-lay and peak-laying stages.** (A) VIP scores and heatmap of differential metabolites among onset-of-lay groups (F31, FC, FH, and FA). (B) VIP scores and heatmap of differential metabolites among peak-laying groups (G31, GC, GH, and GA). (C, D) KEGG pathway enrichment analysis of differential metabolites in onset-of-lay and peak-laying groups, respectively. (E, F) Heatmaps of representative metabolite subclasses associated with population- and stage-associated metabolic variation.

Notably, lysophosphatidylcholine (LPC) and lysophosphatidic acid (LPA), two key intermediates of glycerophospholipid metabolism, showed higher abundance patterns in the commercial populations and Chuqin No. 2 (Fig. 5F). LPC and LPA participate in phospholipid remodeling, lipid transport, and triacylglycerol biosynthesis, thereby linking membrane lipid turnover with neutral lipid deposition (Hishikawa et al., 2008; Yung et al., 2014). Their enrichment was consistent with the enhanced phospholipid remodeling and triacylglycerol accumulation identified in the lipidomic analysis, indicating greater capacity for lipid precursor allocation in these chicken populations. In addition, differences in carnitine-related metabolites suggest variation in fatty acid transport and mitochondrial lipid utilization among chicken populations (Victor et al., 2024). Collectively, these findings indicate population-associated differences in metabolite transport and glycerophospholipid-related metabolism at the 180-day stage. Together with the lipidomic results, variation in LPC/LPA-related metabolites suggests differences in lipid precursor allocation among chicken populations, which may contribute to the observed variation in yolk volatile profiles.

#### 3.4.2 Stage-associated metabolic remodeling related to precursor biosynthesis

In addition to population-associated metabolic variation, sampling stage was associated with extensive remodeling of the yolk metabolome (Fig. 6A-D and Fig. S7). Factorial mixed-effects analysis identified 275 metabolites with significant sampling-stage effects after FDR correction (Table S6). Model-based simple-effect analysis further identified 59, 57, 14, and 64 metabolites showing significant stage-associated differences in populations A, C, H, and 31, respectively. These results indicated a broad stage-associated metabolic shift across chicken populations, although the number and identity of altered metabolites varied among populations. KEGG enrichment analysis consistently identified the pentose phosphate pathway (PPP) among the major pathways associated with stage comparisons (Fig. 6E-H). Higher abundances of PPP-related intermediates, including ribose-5-phosphate and ribulose-5-phosphate, were observed during the peak-laying in several populations (Fig. 6I-L and Fig. S7). As a major source of NADPH and biosynthetic intermediates, the PPP supports reductive biosynthesis and redox homeostasis (Rashida & Laxman, 2021; TeSlaa et al., 2023). In parallel, enrichment of glycolysis and central carbon metabolism was accompanied by changes in carbon intermediates and amino acid-derived metabolites, consistent with altered biosynthetic metabolism between the two sampling stages (Bai et al., 2025).

**Fig. 6:**
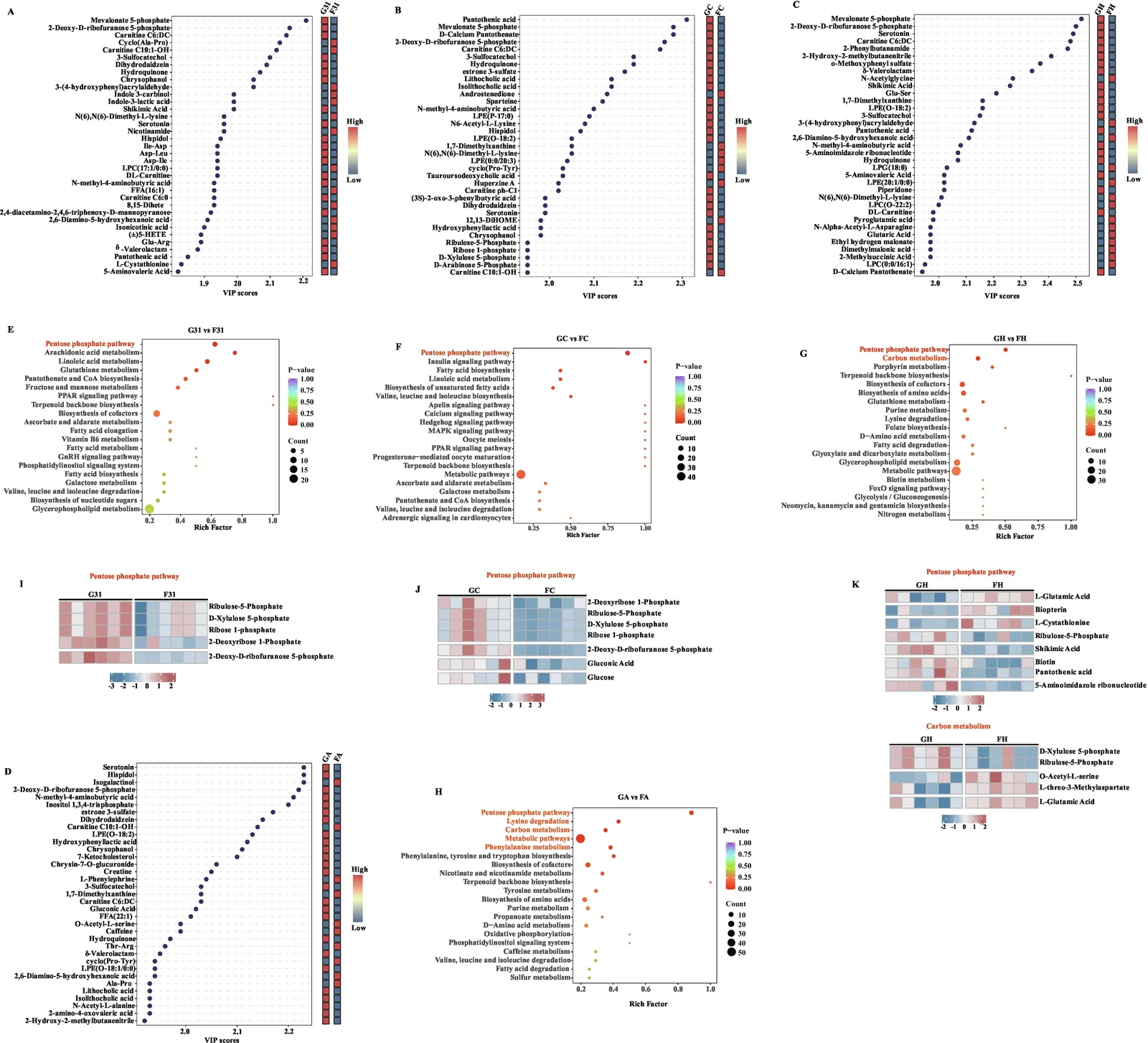
**Stage-associated metabolic remodeling in egg yolks across different chicken populations.** (A-D) VIP scores and heatmaps of differential metabolites between peak-laying and onset-of-lay stages in Chuqin No. 2 (G31 vs. F31), white broilers (GC vs. FC), high-yield layers (GH vs. FH), and local chickens (GA vs. FA), respectively. (E-H) KEGG pathway enrichment analysis of differential metabolites between laying stages. (I-L) Heatmaps of representative metabolites involved in significantly enriched metabolic pathways.

Consistent with these metabolic changes, glycerophospholipid metabolism was also significantly enriched during laying progression, accompanied by increased abundances of lysophosphatidylcholine (LPC)- and lysophosphatidic acid (LPA)-related metabolites (Fig. S7). As intermediates linking phospholipid remodeling with glycerolipid biosynthesis, LPC and LPA are involved in lipid transport, remodeling, and storage (Zhang et al., 2021). Their abundance patterns were consistent with the phospholipid remodeling and triacylglycerol remodeling observed in the lipidomic analysis, indicating coordinated stage-associated changes in carbon and lipid metabolism. Although 17 metabolites showed significant population × stage interactions after FDR correction, the overall sampling-stage effect involved a substantially broader range of metabolites. Together, these findings suggest that the transition between the two sampling stages was accompanied by broad changes in carbon metabolism, phospholipid-related metabolism, and precursor-associated metabolic pathways, providing metabolic support for the stage-associated lipid remodeling observed in egg yolks.

### 3.5 Integrated multi-omics analysis reveals metabolic associations related to egg yolk

#### 3.5.1 Correlation analysis reveals stage-associated associations between lipid remodeling and volatile profiles

To further investigate the associations between lipid remodeling and odor-relevant volatile profiles, Pearson correlation analysis was performed between differential lipid species and odor-relevant volatile compounds at the two sampling stages (Fig. 7A-B). The resulting correlation networks revealed stage-associated changes in lipid–volatile relationships, suggesting that the lipid–volatile association patterns differed between sampling stages. At the onset-of-lay stage (Fig. 7A), several odor-relevant aldehydes, particularly 2-undecenal and (E)-2-undecenal, showed strong positive correlations with phosphatidylethanolamine (PE)- and phosphatidylcholine (PC)-containing lipid species, including PE(18:0_18:1) and PC(16:0_18:1). These phospholipids contain abundant unsaturated fatty acids and may represent oxidation-sensitive lipid reservoirs. Oxidative degradation of PUFA-containing phospholipids has been reported to generate aldehydic volatile compounds, including 2-undecenal-related compounds (Lin & Blank, 2003), supporting a potential contribution of membrane phospholipid composition to aroma precursor availability during early laying.

**Fig. 7:**
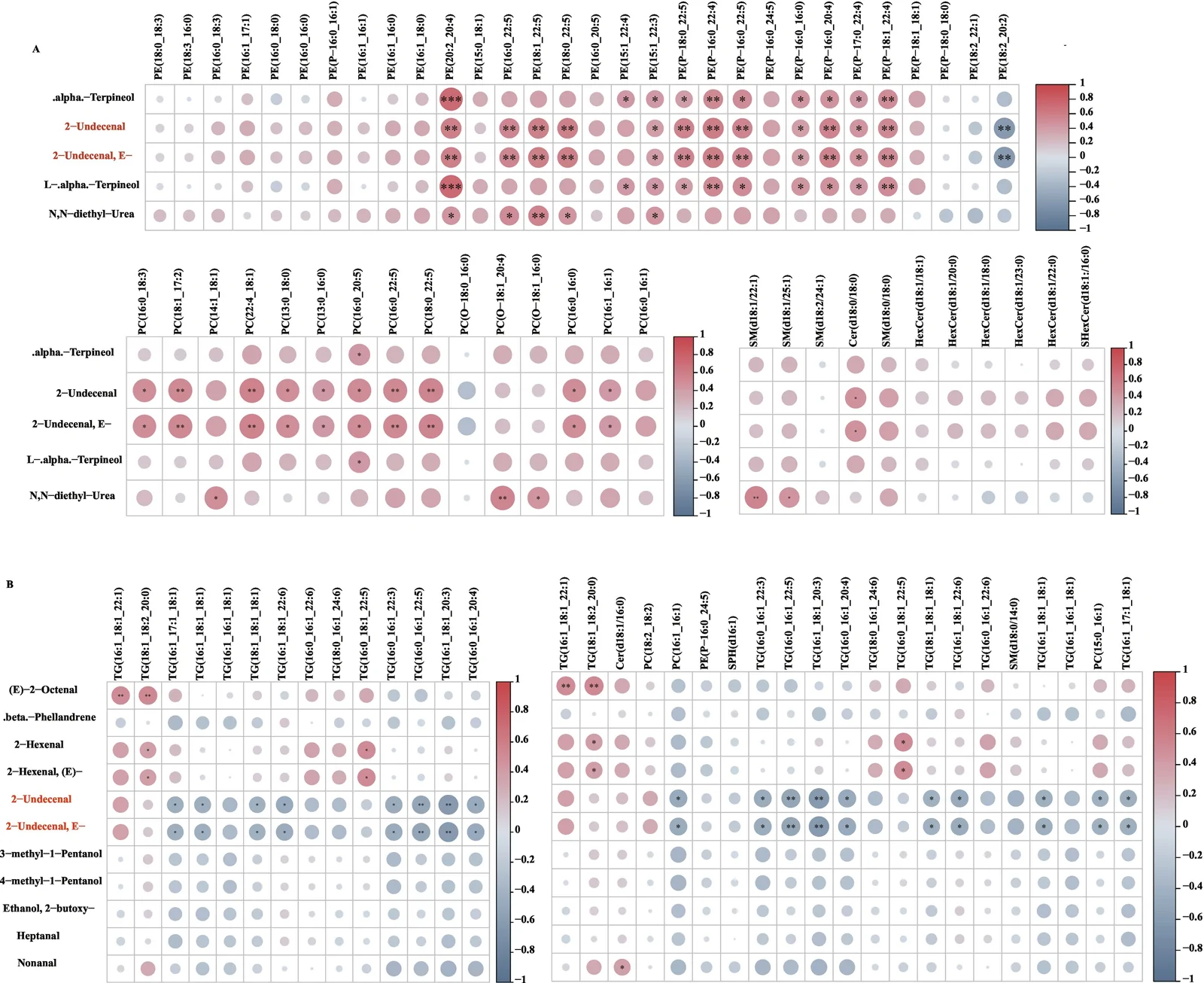
**Integrated lipid–volatile association networks reveal associations between lipid remodeling and egg yolk volatile profiles.** (A) Pearson correlation networks between differential volatile compounds and lipid species during the onset-of-lay stage. (B) Pearson correlation networks between differential volatile compounds and lipid species during the peak-laying stage. Correlation significance is indicated as *p < 0.05, **p < 0.01, and ***p < 0.001.

At the peak-laying stage (Fig. 7B), the lipid–volatile interaction network showed a different organization. Correlations between several aldehydes and triacylglycerol (TG) species were generally weaker, whereas positive associations with PUFA-containing PC species remained evident. For example, 2-undecenal isomers showed negative correlations with several long-chain TG species, including TG(16:1_18:1_22:1), indicating that neutral lipid abundance alone did not account for aldehyde variation. In addition, nonanal was positively correlated with several sphingomyelin species, including SM(d18:0/18:0), suggesting an association between sphingolipid composition and volatile precursor availability. Overall, PUFA-containing membrane phospholipids showed consistent associations with odor-relevant aldehydes across the two sampling stages. These findings support membrane phospholipid remodeling as an important lipidomic feature associated with variation in egg yolk volatile profiles.

#### 3.5.2 Integrated metabolite–volatile associations reveal metabolic networks associated with aroma variation

To further investigate the metabolic processes associated with aroma variation, Pearson correlation analysis was performed between differential metabolites involved in key metabolic pathways and odor-relevant volatile compounds (Fig. S8A—B). The resulting metabolite–volatile network revealed coordinated associations among amino acid metabolism, phospholipid-related metabolism, and lipid-derived volatile profiles, suggesting that multiple metabolic processes are linked to variation in egg yolk aroma.

Amino acid-related metabolites, including branched-chain amino acids and several aromatic amino acid derivatives, showed significant correlations with lipid oxidation-derived aldehydes such as nonanal, heptanal, and 2-undecenal (Fig. S8A). Although amino acids can contribute directly to flavor generation during thermal processing through Strecker degradation and Maillard reactions (Hu et al., 2024), the correlations observed in fresh egg yolks are more likely to reflect coordinated metabolic activity rather than direct volatile production. Amino acid metabolism may therefore contribute indirectly by providing carbon skeletons and metabolic intermediates associated with lipid biosynthesis and precursor formation (Hidalgo & Zamora, 2008). Compared with amino acid metabolites, lysophospholipid-associated metabolites showed closer relationships with several odor-relevant volatiles (Fig. S8B). LPC(O-20:0), for example, was positively correlated with nonanal and 2-undecenal, whereas LPE species displayed distinct correlation patterns. As intermediates of phospholipid remodeling, LPCs participate in membrane lipid turnover and lipid transport. Their association with aldehyde abundance suggests that lysophospholipid metabolism may be linked to variation in oxidation-sensitive lipid precursor pools during yolk development.

Integration of the metabolomic, lipidomic, and volatilomic datasets revealed coordinated associations between primary metabolism, lipid remodeling, and yolk volatile profiles. Carbon and amino acid metabolism were associated with potential precursor supply, whereas phospholipid- and lysophospholipid-related pools were closely linked to the availability of oxidation-sensitive lipid substrates. Oxidative conversion of these lipid substrates may contribute to the formation of odor-relevant aldehydes, including heptanal, nonanal, and (E)-2-octenal. Rather than indicating a single dominant pathway, these results suggest that yolk volatile variation is associated with coordinated changes in primary metabolism and lipid remodeling. Previous multi-omics studies have characterized flavor-related molecular differences among poultry eggs from different genetic or production backgrounds (Yang et al., 2023). For example, Yang et al. (2023) integrated volatilomics, metabolomics, and lipidomics to characterize flavor differences between Taihe black-boned silky fowl and Hy-line Brown egg yolks at a single sampling stage. In the present study, four chicken populations were examined at two laying stages within the same analytical framework, allowing population-associated variation to be evaluated in the context of laying progression. The metabolic features associated with yolk volatile composition differed between the two sampling stages: population-associated differences were evident at both stages, whereas broader stage-associated remodeling involved phospholipids, neutral lipids, and central carbon metabolism. Thus, the metabolic context associated with yolk volatile variation changes during laying progression, with corresponding variation in potential lipid precursor pools.

## 4 Conclusion

This study integrated volatilomics, lipidomics, and metabolomics to characterize population- and stage-associated variation in egg yolk aroma and its associated metabolic features. Factorial analyses showed that sampling stage was associated with broader changes across the yolk volatilome, lipidome, and metabolome, whereas population-associated effects were more selective. The later sampling stage was characterized by altered abundance patterns of lipid oxidation-derived aldehydes, together with extensive remodeling of phospholipids, neutral lipids, and central carbon metabolism. Integrated multi-omics analysis further revealed coordinated associations among carbon metabolism, lipid precursor allocation, phospholipid remodeling, and volatile profiles. In particular, PUFA-containing glycerophospholipids and lysophospholipid-related metabolites were closely associated with several odor-relevant aldehydes, suggesting an association between membrane lipid remodeling and potential volatile precursor availability. Population-associated differences in specific lipid and metabolite pools were superimposed on the broader stage-associated metabolic remodeling, indicating that yolk precursor composition varies across both chicken populations and laying progression. Overall, these findings provide a metabolic framework for understanding population- and stage-associated variation in egg yolk aroma and provide a basis for future studies on the regulation of egg flavor quality.

## CRediT authorship contribution statement

**Cui Ma:** Writing – original draft, Validation, Investigation, Formal analysis, Data curation. **Jinsong Pi:** Visualization, Validation. **Ailuan Pan:** Supervision. **Yaru Chen:** Supervision. **Ming Fu:** Supervision. **Tao Huang:** Supervision. **Hao Zhang:** Supervision. **Yan Wu:** Writing – review & editing, Supervision, Resources, Project administration, Funding acquisition.

## Declaration of competing interest

The authors declare that they have no known competing financial interests or personal relationships that could have appeared to influence the work reported in this paper.

## Data Availability

Data will be made available on request.
