# Supplementary material for: Integrated multi-omics reveals lipid remodeling underlying population- and stage-associated aroma variation in chicken egg yolks
Source: Food Chem X. 2026 Oct 1;39:104548. doi: 10.1016/j.fochx.2026.104548 (PMC13671372; doi:10.1016/j.fochx.2026.104548)
Supplement: Supplementary file 1 [file mmc1.pdf]

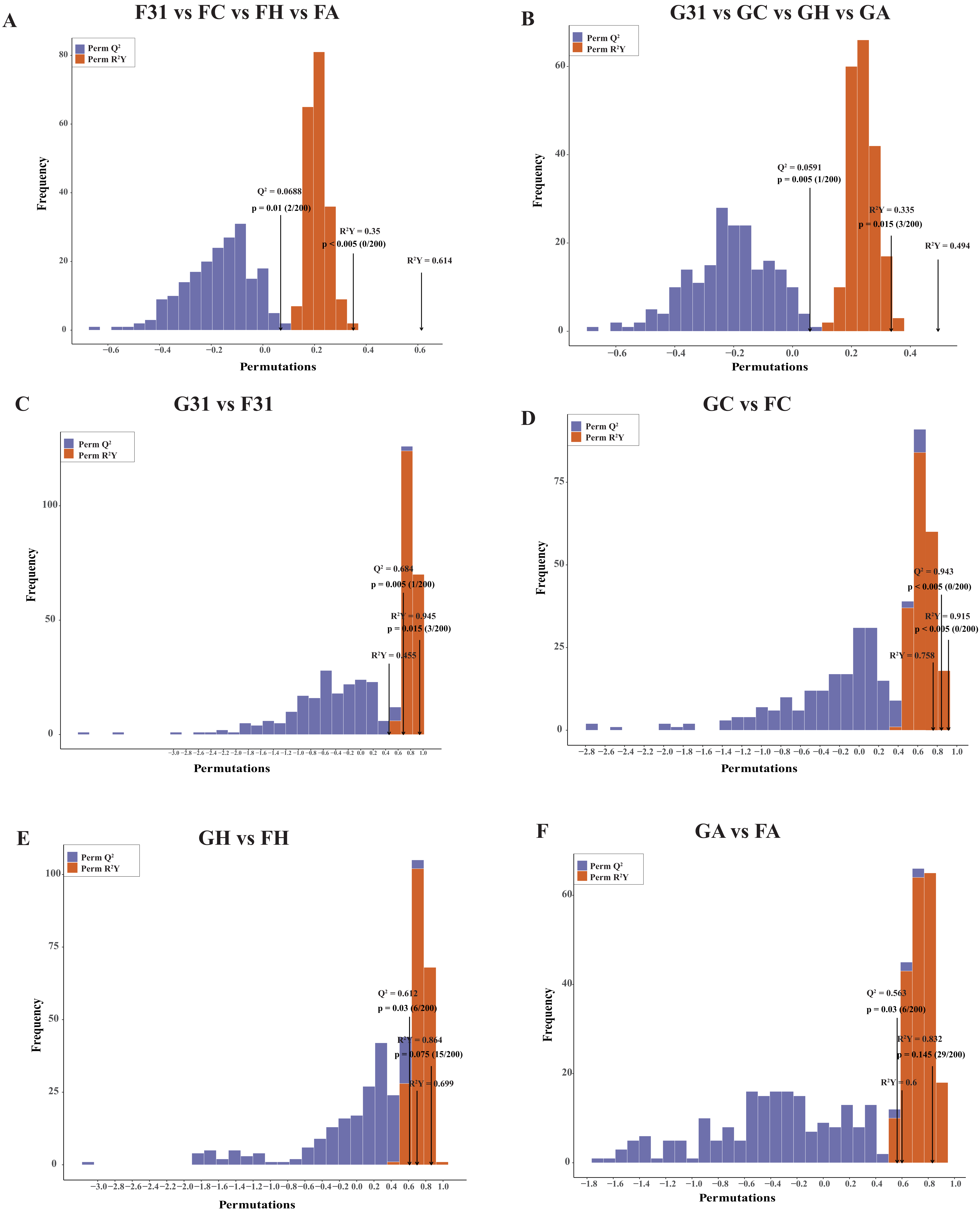

A
F31 vs FC vs FH vs FA
Perm Q²
Perm R²Y
Frequency
Permutations
Q² = 0.0688
p = 0.01 (2/200)
R²Y = 0.35
p < 0.005 (0/200)
R²Y = 0.614
B
G31 vs GC vs GH vs GA
Perm Q²
Perm R²Y
Frequency
Permutations
Q² = 0.0591
p = 0.005 (1/200)
R²Y = 0.335
p = 0.015 (3/200)
R²Y = 0.494
C
G31 vs F31
Perm Q²
Perm R²Y
Frequency
Permutations
Q² = 0.684
p = 0.005 (1/200)
R²Y = 0.945
p = 0.015 (3/200)
R²Y = 0.455
D
GC vs FC
Perm Q²
Perm R²Y
Frequency
Permutations
Q² = 0.943
p < 0.005 (0/200)
R²Y = 0.915
p < 0.005 (0/200)
R²Y = 0.758
E
GH vs FH
Perm Q²
Perm R²Y
Frequency
Permutations
Q² = 0.612
p = 0.03 (6/200)
R²Y = 0.864
p = 0.075 (15/200)
R²Y = 0.699
F
GA vs FA
Perm Q²
Perm R²Y
Frequency
Permutations
Q² = 0.563
p = 0.03 (6/200)
R²Y = 0.832
p = 0.145 (29/200)
R²Y = 0.6
