# Supplementary material for: Integrated multi-omics reveals lipid remodeling underlying population- and stage-associated aroma variation in chicken egg yolks
Source: Food Chem X. 2026 Oct 1;39:104548. doi: 10.1016/j.fochx.2026.104548 (PMC13671372; doi:10.1016/j.fochx.2026.104548)
Supplement: Supplementary file 2 [file mmc2.pdf]

A

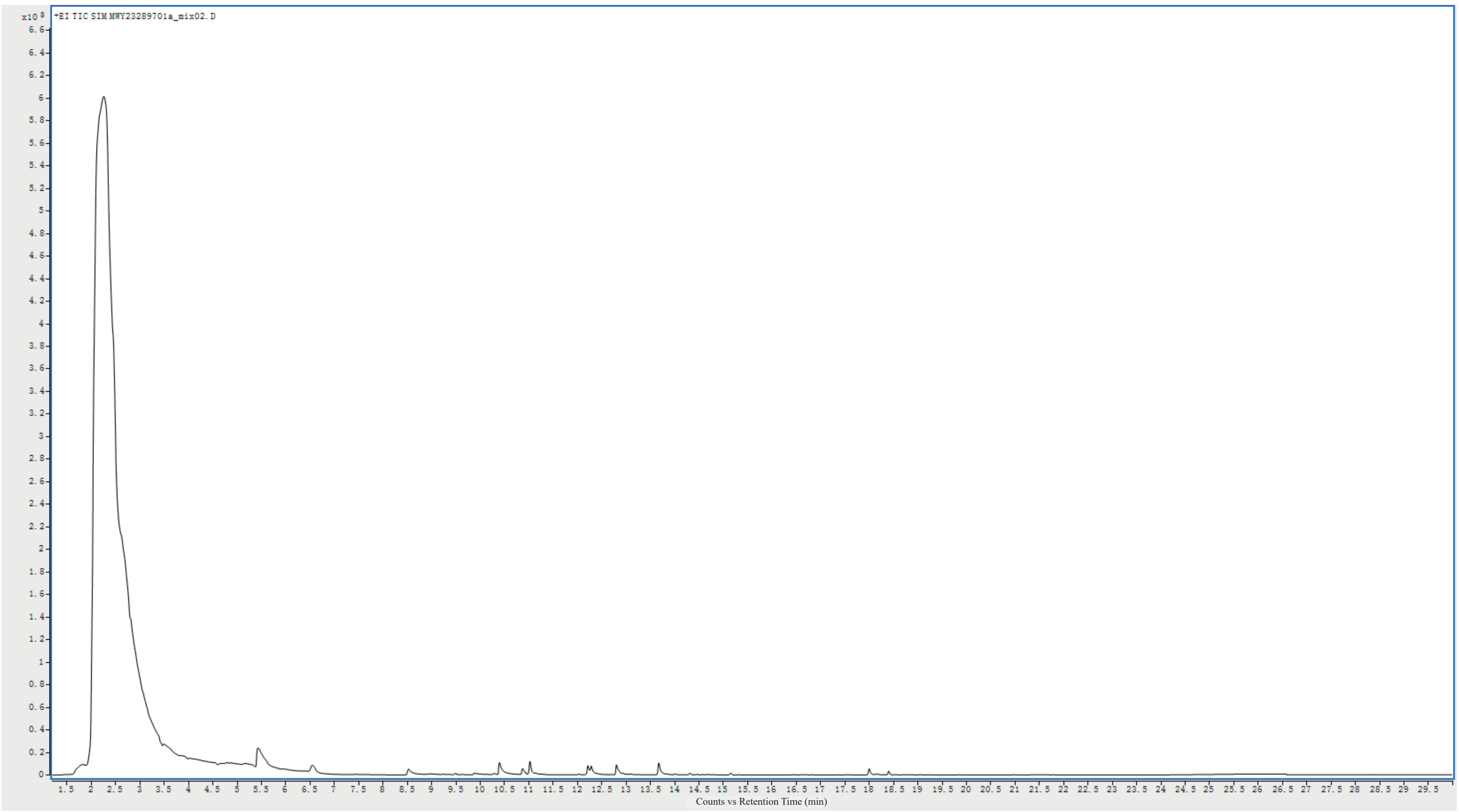

Counts vs Retention Time (min)

B

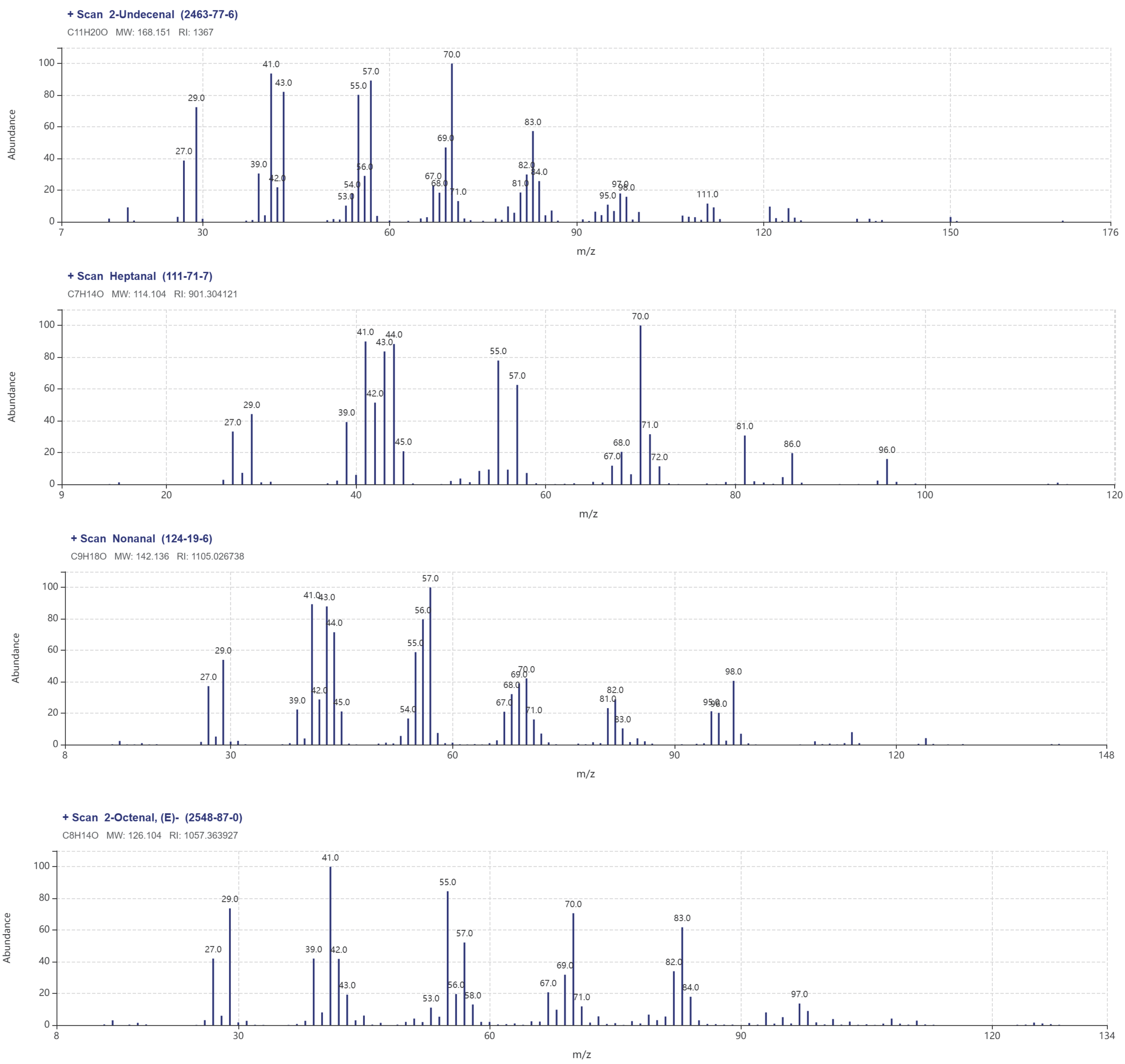

+ Scan 2-Undecenal (2463-77-6)
C11H20O MW: 168.151 RI: 1367
+ Scan Heptanal (111-71-7)
C7H14O MW: 114.104 RI: 901.304121
+ Scan Nonanal (124-19-6)
C9H18O MW: 142.136 RI: 1105.026738
+ Scan 2-Octenal, (E)- (2548-87-0)
C8H14O MW: 126.104 RI: 1057.363927
Abundance
m/z
