# Supplementary material for: Integrated multi-omics reveals lipid remodeling underlying population- and stage-associated aroma variation in chicken egg yolks
Source: Food Chem X. 2026 Oct 1;39:104548. doi: 10.1016/j.fochx.2026.104548 (PMC13671372; doi:10.1016/j.fochx.2026.104548)
Supplement: Supplementary file 4 [file mmc4.pdf]

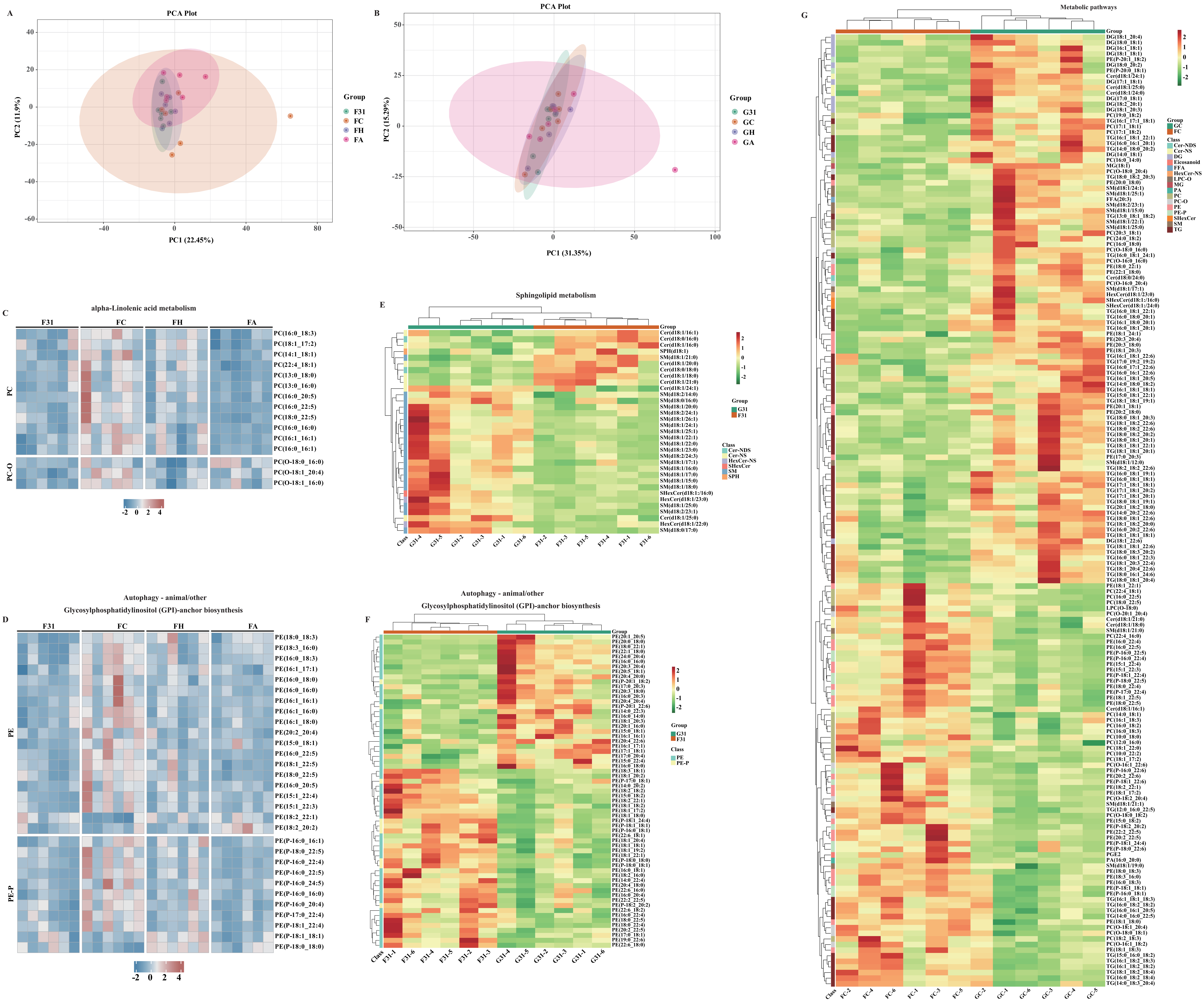

A
PCA Plot
PC1 (22.45%)
PC2 (11.9%)
Group
F31
FC
FH
FA
B
PCA Plot
PC1 (31.35%)
PC2 (15.29%)
Group
G31
GC
GH
GA
C
alpha-Linolenic acid metabolism
F31
FC
FH
FA
PC
PC-O
PC(16:0_18:3)
PC(18:1_17:2)
PC(14:1_18:1)
PC(22:4_18:1)
PC(13:0_18:0)
PC(13:0_16:0)
PC(16:0_20:5)
PC(16:0_22:5)
PC(18:0_22:5)
PC(16:0_16:0)
PC(16:1_16:1)
PC(16:0_16:1)
PC(O-18:0_16:0)
PC(O-18:1_20:4)
PC(O-18:1_16:0)
D
Autophagy - animal/other
Glycosylphosphatidylinositol (GPI)-anchor biosynthesis
F31
FC
FH
FA
PE
PE-P
PE(18:0_18:3)
PE(18:3_16:0)
PE(16:0_18:3)
PE(16:1_17:1)
PE(16:0_18:0)
PE(16:0_16:0)
PE(16:1_16:1)
PE(16:1_16:0)
PE(16:1_18:0)
PE(20:2_20:4)
PE(15:0_18:1)
PE(16:0_22:5)
PE(18:1_22:5)
PE(18:0_22:5)
PE(16:0_20:5)
PE(15:1_22:4)
PE(15:1_22:3)
PE(18:2_22:1)
PE(18:2_20:2)
PE(P-16:0_16:1)
PE(P-18:0_22:5)
PE(P-16:0_22:4)
PE(P-16:0_22:5)
PE(P-16:0_24:5)
PE(P-16:0_16:0)
PE(P-16:0_20:4)
PE(P-17:0_22:4)
PE(P-18:1_22:4)
PE(P-18:1_18:1)
PE(P-18:0_18:0)
E
Sphingolipid metabolism
Group
Class
Cer-NDS
Cer-NS
HexCer-NS
SHexCer
SM
SPH
G31
F31
G31-4
G31-5
G31-2
G31-3
G31-1
G31-6
F31-2
F31-3
F31-5
F31-4
F31-1
F31-6
F
Autophagy - animal/other
Glycosylphosphatidylinositol (GPI)-anchor biosynthesis
Group
Class
PE
PE-P
G31
F31
F31-1
F31-6
F31-4
F31-5
F31-2
F31-3
G31-4
G31-5
G31-2
G31-3
G31-1
G31-6
G
Metabolic pathways
Group
GC
FC
Class
Cer-NDS
Cer-NS
DG
Eicosanoid
FFA
HexCer-NS
LPC-O
MG
PA
PC
PC-O
PE
PE-P
SHexCer
SM
TG
FC-2
FC-4
FC-6
FC-1
FC-3
FC-5
GC-2
GC-1
GC-6
GC-3
GC-4
GC-5
