# Supplementary material for: Integrated multi-omics reveals lipid remodeling underlying population- and stage-associated aroma variation in chicken egg yolks
Source: Food Chem X. 2026 Oct 1;39:104548. doi: 10.1016/j.fochx.2026.104548 (PMC13671372; doi:10.1016/j.fochx.2026.104548)
Supplement: Supplementary file 5 [file mmc5.pdf]

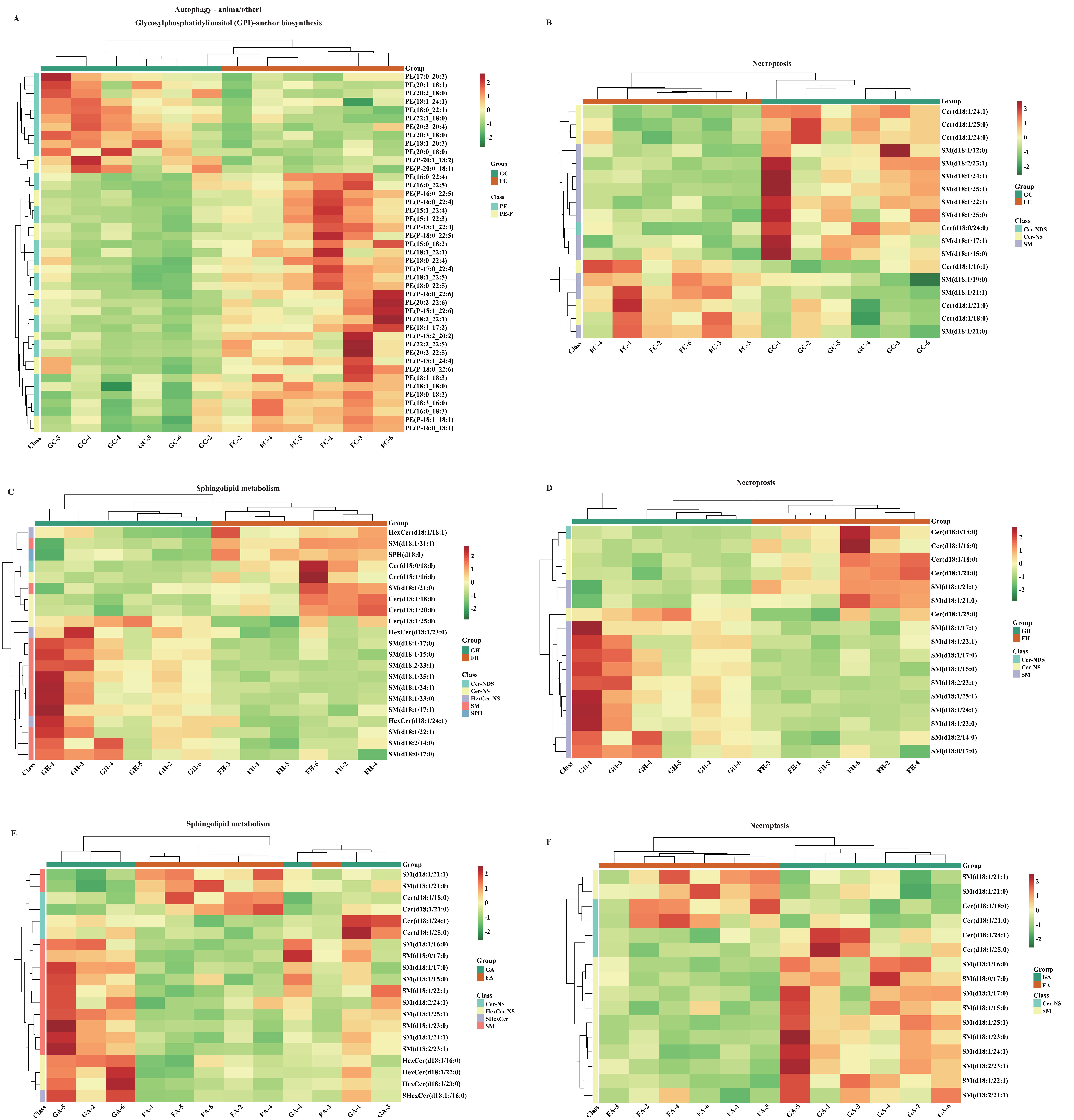
A
Autophagy - anima/otherl
Glycosylphosphatidylinositol (GPI)-anchor biosynthesis
Group
PE(17:0_20:3)
PE(20:1_18:1)
PE(20:2_18:0)
PE(18:1_24:1)
PE(18:0_22:1)
PE(22:1_18:0)
PE(20:3_20:4)
PE(20:3_18:0)
PE(18:1_20:3)
PE(20:0_18:0)
PE(P-20:1_18:2)
PE(P-20:0_18:1)
PE(16:0_22:4)
PE(16:0_22:5)
PE(P-16:0_22:5)
PE(P-16:0_22:4)
PE(15:1_22:4)
PE(15:1_22:3)
PE(P-18:1_22:4)
PE(P-18:0_22:5)
PE(15:0_18:2)
PE(18:1_22:1)
PE(18:0_22:4)
PE(P-17:0_22:4)
PE(18:1_22:5)
PE(18:0_22:5)
PE(P-16:0_22:6)
PE(20:2_22:6)
PE(P-18:1_22:6)
PE(18:2_22:1)
PE(18:1_17:2)
PE(P-18:2_20:2)
PE(22:2_22:5)
PE(20:2_22:5)
PE(P-18:1_24:4)
PE(P-18:0_22:6)
PE(18:1_18:3)
PE(18:1_18:0)
PE(18:0_18:3)
PE(18:3_16:0)
PE(16:0_18:3)
PE(P-18:1_18:1)
PE(P-16:0_18:1)
Class
GC-3
GC-4
GC-1
GC-5
GC-6
GC-2
FC-2
FC-4
FC-5
FC-1
FC-3
FC-6
2
1
0
-1
-2
Group
GC
FC
Class
PE
PE-P
B
Necroptosis
Group
Cer(d18:1/24:1)
Cer(d18:1/25:0)
Cer(d18:1/24:0)
SM(d18:1/12:0)
SM(d18:2/23:1)
SM(d18:1/24:1)
SM(d18:1/25:1)
SM(d18:1/22:1)
SM(d18:1/25:0)
Cer(d18:0/24:0)
SM(d18:1/17:1)
SM(d18:1/15:0)
Cer(d18:1/16:1)
SM(d18:1/19:0)
SM(d18:1/21:1)
Cer(d18:1/21:0)
Cer(d18:1/18:0)
SM(d18:1/21:0)
Class
FC-4
FC-1
FC-2
FC-6
FC-3
FC-5
GC-1
GC-2
GC-5
GC-4
GC-3
GC-6
2
1
0
-1
-2
Group
GC
FC
Class
Cer-NDS
Cer-NS
SM
C
Sphingolipid metabolism
Group
HexCer(d18:1/18:1)
SM(d18:1/21:1)
SPH(d18:0)
Cer(d18:0/18:0)
Cer(d18:1/16:0)
SM(d18:1/21:0)
Cer(d18:1/18:0)
Cer(d18:1/20:0)
Cer(d18:1/25:0)
HexCer(d18:1/23:0)
SM(d18:1/17:0)
SM(d18:1/15:0)
SM(d18:2/23:1)
SM(d18:1/25:1)
SM(d18:1/24:1)
SM(d18:1/23:0)
SM(d18:1/17:1)
HexCer(d18:1/24:1)
SM(d18:1/22:1)
SM(d18:2/14:0)
SM(d18:0/17:0)
Class
GH-1
GH-3
GH-4
GH-5
GH-2
GH-6
FH-3
FH-1
FH-5
FH-6
FH-2
FH-4
2
1
0
-1
-2
Group
GH
FH
Class
Cer-NDS
Cer-NS
HexCer-NS
SM
SPH
D
Necroptosis
Group
Cer(d18:0/18:0)
Cer(d18:1/16:0)
Cer(d18:1/18:0)
Cer(d18:1/20:0)
SM(d18:1/21:1)
SM(d18:1/21:0)
Cer(d18:1/25:0)
SM(d18:1/17:1)
SM(d18:1/22:1)
SM(d18:1/17:0)
SM(d18:1/15:0)
SM(d18:2/23:1)
SM(d18:1/25:1)
SM(d18:1/24:1)
SM(d18:1/23:0)
SM(d18:2/14:0)
SM(d18:0/17:0)
Class
GH-1
GH-3
GH-4
GH-5
GH-2
GH-6
FH-3
FH-1
FH-5
FH-6
FH-2
FH-4
2
1
0
-1
-2
Group
GH
FH
Class
Cer-NDS
Cer-NS
SM
E
Sphingolipid metabolism
Group
SM(d18:1/21:1)
SM(d18:1/21:0)
Cer(d18:1/18:0)
Cer(d18:1/21:0)
Cer(d18:1/24:1)
Cer(d18:1/25:0)
SM(d18:1/16:0)
SM(d18:0/17:0)
SM(d18:1/17:0)
SM(d18:1/15:0)
SM(d18:1/22:1)
SM(d18:2/24:1)
SM(d18:1/25:1)
SM(d18:1/23:0)
SM(d18:1/24:1)
SM(d18:2/23:1)
HexCer(d18:1/16:0)
HexCer(d18:1/22:0)
HexCer(d18:1/23:0)
SHexCer(d18:1:/16:0)
Class
GA-5
GA-2
GA-6
FA-1
FA-5
FA-6
FA-2
FA-4
GA-4
FA-3
GA-1
GA-3
2
1
0
-1
-2
Group
GA
FA
Class
Cer-NS
HexCer-NS
SHexCer
SM
F
Necroptosis
Group
SM(d18:1/21:1)
SM(d18:1/21:0)
Cer(d18:1/18:0)
Cer(d18:1/21:0)
Cer(d18:1/24:1)
Cer(d18:1/25:0)
SM(d18:1/16:0)
SM(d18:0/17:0)
SM(d18:1/17:0)
SM(d18:1/15:0)
SM(d18:1/25:1)
SM(d18:1/23:0)
SM(d18:1/24:1)
SM(d18:2/23:1)
SM(d18:1/22:1)
SM(d18:2/24:1)
Class
FA-3
FA-2
FA-4
FA-6
FA-1
FA-5
GA-5
GA-1
GA-3
GA-4
GA-2
GA-6
2
1
0
-1
-2
Group
GA
FA
Class
Cer-NS
SM
