# Supplementary material for: Integrated multi-omics reveals lipid remodeling underlying population- and stage-associated aroma variation in chicken egg yolks
Source: Food Chem X. 2026 Oct 1;39:104548. doi: 10.1016/j.fochx.2026.104548 (PMC13671372; doi:10.1016/j.fochx.2026.104548)
Supplement: Supplementary file 6 [file mmc6.pdf]

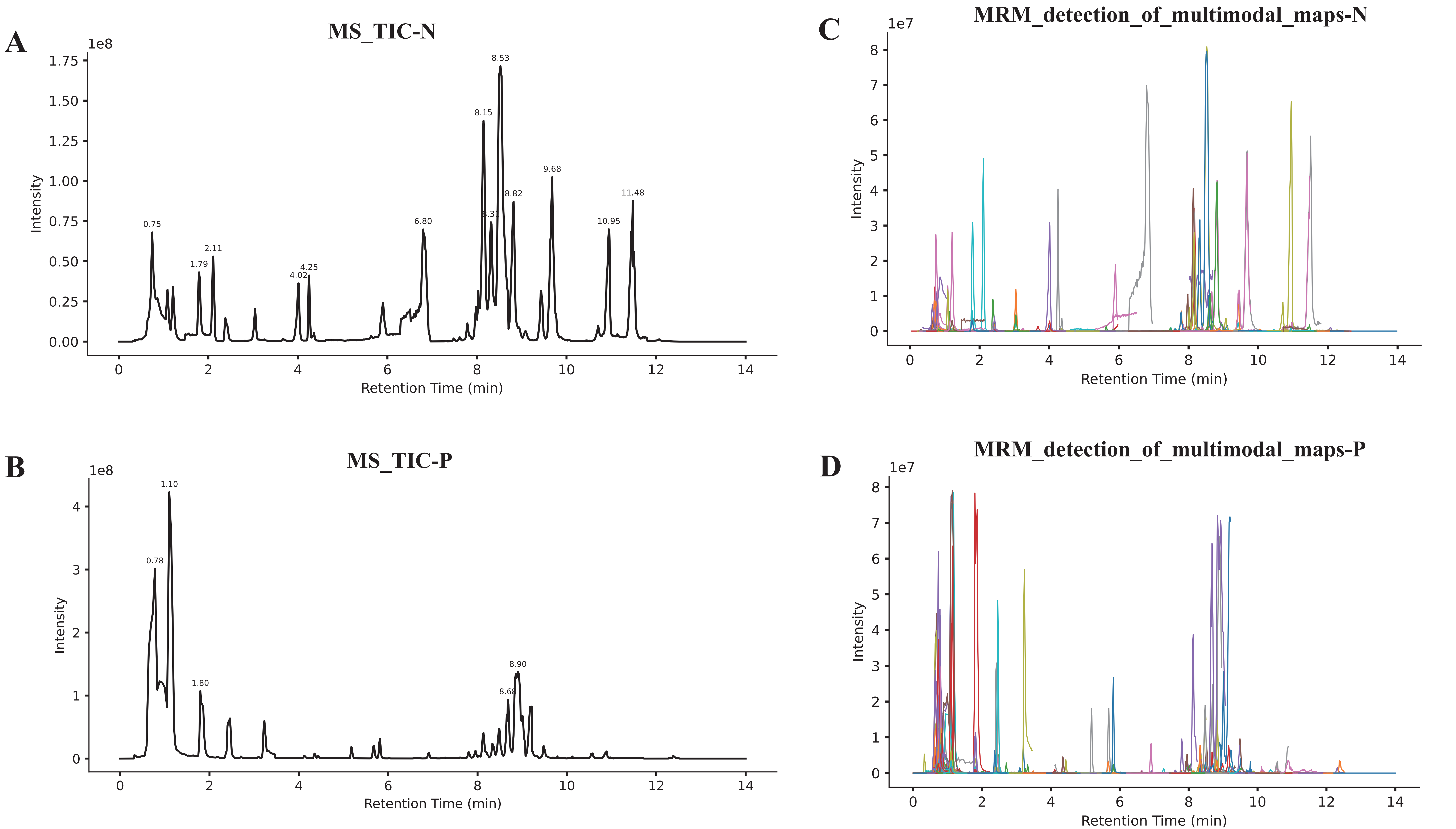

A
MS_TIC-N
1e8
Intensity
Retention Time (min)
0.75
1.79
2.11
4.02
4.25
6.80
8.15
8.31
8.53
8.82
9.68
10.95
11.48
B
MS_TIC-P
1e8
Intensity
Retention Time (min)
0.78
1.10
1.80
8.68
8.90
C
MRM_detection_of_multimodal_maps-N
1e7
Intensity
Retention Time (min)
D
MRM_detection_of_multimodal_maps-P
1e7
Intensity
Retention Time (min)
