# Supplementary material for: Integrated multi-omics reveals lipid remodeling underlying population- and stage-associated aroma variation in chicken egg yolks
Source: Food Chem X. 2026 Oct 1;39:104548. doi: 10.1016/j.fochx.2026.104548 (PMC13671372; doi:10.1016/j.fochx.2026.104548)
Supplement: Supplementary file 7 [file mmc7.pdf]

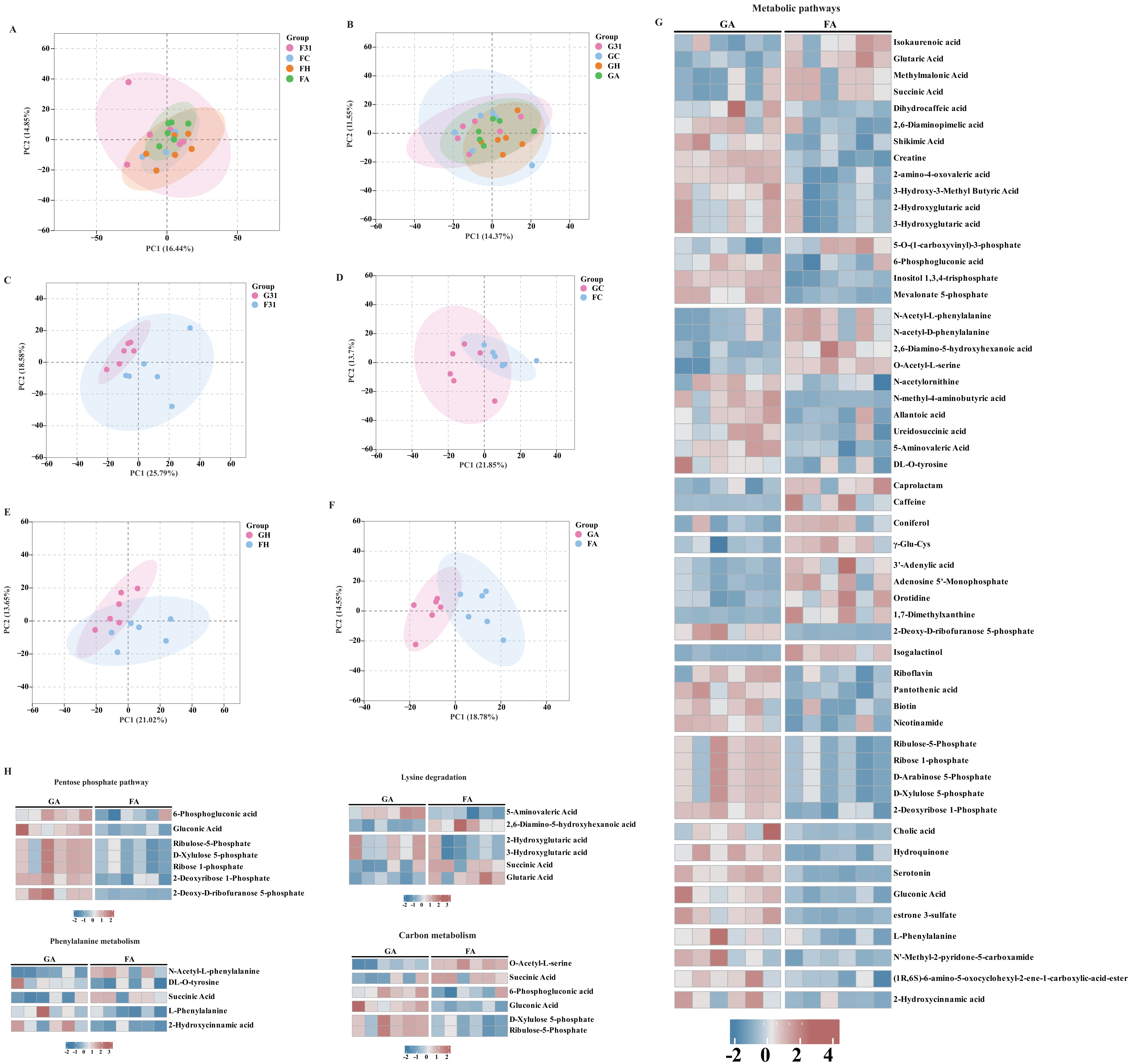

A
Group
F31
FC
FH
FA
PC1 (16.44%)
PC2 (14.85%)
B
Group
G31
GC
GH
GA
PC1 (14.37%)
PC2 (11.55%)
C
Group
G31
F31
PC1 (25.79%)
PC2 (18.58%)
D
Group
GC
FC
PC1 (21.85%)
PC2 (13.7%)
E
Group
GH
FH
PC1 (21.02%)
PC2 (13.65%)
F
Group
GA
FA
PC1 (18.78%)
PC2 (14.55%)
G
Metabolic pathways
GA
FA
Isokaurenoic acid
Glutaric Acid
Methylmalonic Acid
Succinic Acid
Dihydrocaffeic acid
2,6-Diaminopimelic acid
Shikimic Acid
Creatine
2-amino-4-oxovaleric acid
3-Hydroxy-3-Methyl Butyric Acid
2-Hydroxyglutaric acid
3-Hydroxyglutaric acid
5-O-(1-carboxyvinyl)-3-phosphate
6-Phosphogluconic acid
Inositol 1,3,4-trisphosphate
Mevalonate 5-phosphate
N-Acetyl-L-phenylalanine
N-acetyl-D-phenylalanine
2,6-Diamino-5-hydroxyhexanoic acid
O-Acetyl-L-serine
N-acetylornithine
N-methyl-4-aminobutyric acid
Allantoic acid
Ureidosuccinic acid
5-Aminovaleric Acid
DL-O-tyrosine
Caprolactam
Caffeine
Coniferol
γ-Glu-Cys
3'-Adenylic acid
Adenosine 5'-Monophosphate
Orotidine
1,7-Dimethylxanthine
2-Deoxy-D-ribofuranose 5-phosphate
Isogalactinol
Riboflavin
Pantothenic acid
Biotin
Nicotinamide
Ribulose-5-Phosphate
Ribose 1-phosphate
D-Arabinose 5-Phosphate
D-Xylulose 5-phosphate
2-Deoxyribose 1-Phosphate
Cholic acid
Hydroquinone
Serotonin
Gluconic Acid
estrone 3-sulfate
L-Phenylalanine
N'-Methyl-2-pyridone-5-carboxamide
(1R,6S)-6-amino-5-oxocyclohexyl-2-ene-1-carboxylic-acid-ester
2-Hydroxycinnamic acid
-2 0 2 4
H
Pentose phosphate pathway
GA
FA
6-Phosphogluconic acid
Gluconic Acid
Ribulose-5-Phosphate
D-Xylulose 5-phosphate
Ribose 1-phosphate
2-Deoxyribose 1-Phosphate
2-Deoxy-D-ribofuranose 5-phosphate
Lysine degradation
5-Aminovaleric Acid
2,6-Diamino-5-hydroxyhexanoic acid
2-Hydroxyglutaric acid
3-Hydroxyglutaric acid
Succinic Acid
Glutaric Acid
Phenylalanine metabolism
N-Acetyl-L-phenylalanine
DL-O-tyrosine
Succinic Acid
L-Phenylalanine
2-Hydroxycinnamic acid
Carbon metabolism
O-Acetyl-L-serine
Succinic Acid
6-Phosphogluconic acid
Gluconic Acid
D-Xylulose 5-phosphate
Ribulose-5-Phosphate
