# Supplementary material for: Integrated multi-omics reveals lipid remodeling underlying population- and stage-associated aroma variation in chicken egg yolks
Source: Food Chem X. 2026 Oct 1;39:104548. doi: 10.1016/j.fochx.2026.104548 (PMC13671372; doi:10.1016/j.fochx.2026.104548)
Supplement: Supplementary file 8 [file mmc8.pdf]

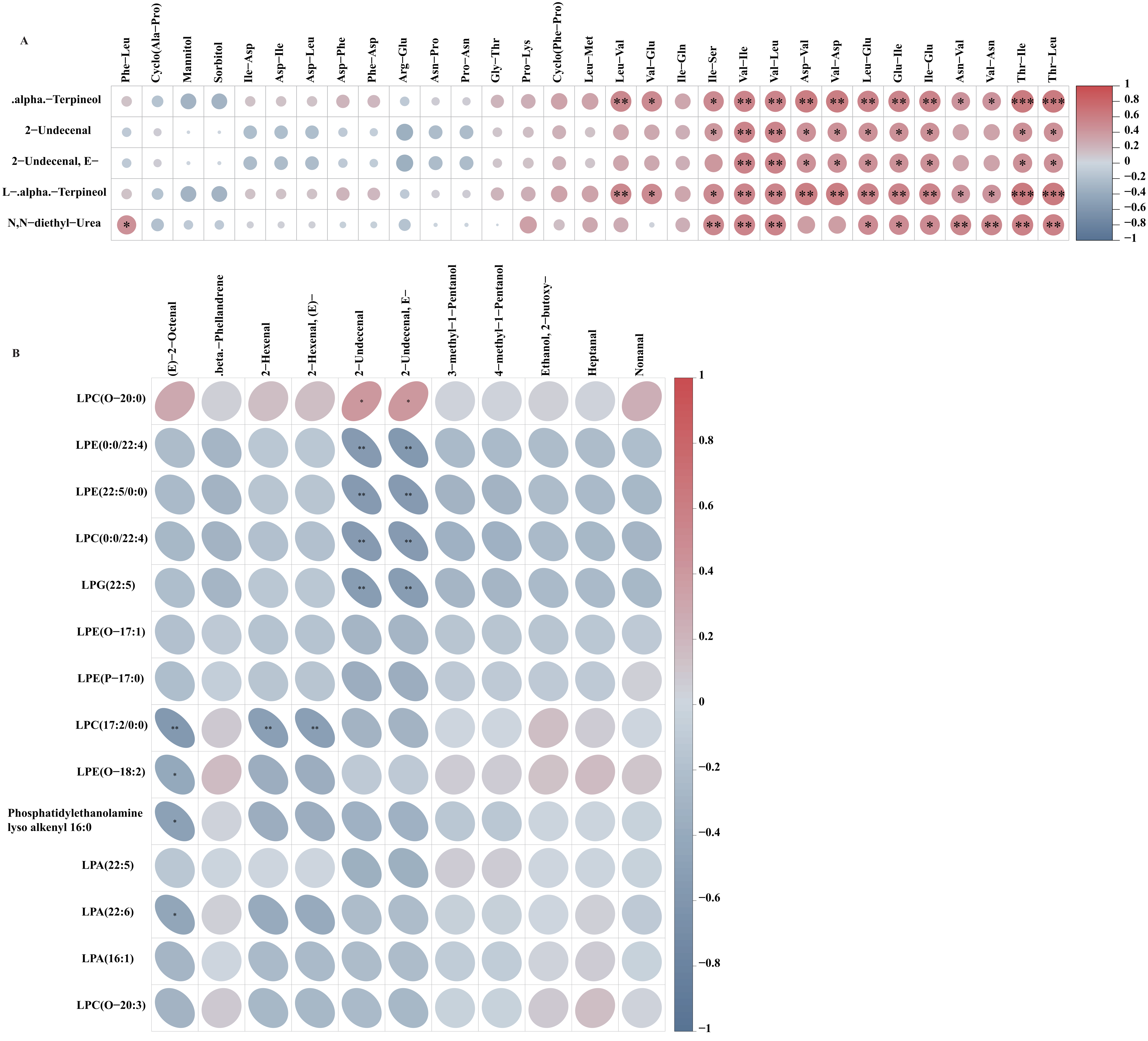
A
Phe−Leu
Cyclo(Ala−Pro)
Mannitol
Sorbitol
Ile−Asp
Asp−Ile
Asp−Leu
Asp−Phe
Phe−Asp
Arg−Glu
Asn−Pro
Pro−Asn
Gly−Thr
Pro−Lys
Cyclo(Phe−Pro)
Leu−Met
Leu−Val
Val−Glu
Ile−Gln
Ile−Ser
Val−Ile
Val−Leu
Asp−Val
Val−Asp
Leu−Glu
Glu−Ile
Ile−Glu
Asn−Val
Val−Asn
Thr−Ile
Thr−Leu
.alpha.−Terpineol
2−Undecenal
2−Undecenal, E−
L−.alpha.−Terpineol
N,N−diethyl−Urea
1
0.8
0.6
0.4
0.2
0
−0.2
−0.4
−0.6
−0.8
−1
B
(E)−2−Octenal
.beta.−Phellandrene
2−Hexenal
2−Hexenal, (E)−
2−Undecenal
2−Undecenal, E−
3−methyl−1−Pentanol
4−methyl−1−Pentanol
Ethanol, 2−butoxy−
Heptanal
Nonanal
LPC(O−20:0)
LPE(0:0/22:4)
LPE(22:5/0:0)
LPC(0:0/22:4)
LPG(22:5)
LPE(O−17:1)
LPE(P−17:0)
LPC(17:2/0:0)
LPE(O−18:2)
Phosphatidylethanolamine lyso alkenyl 16:0
LPA(22:5)
LPA(22:6)
LPA(16:1)
LPC(O−20:3)
1
0.8
0.6
0.4
0.2
0
−0.2
−0.4
−0.6
−0.8
−1
