# Supplementary material for: Integrated multi-omics reveals lipid remodeling underlying population- and stage-associated aroma variation in chicken egg yolks
Source: Food Chem X. 2026 Oct 1;39:104548. doi: 10.1016/j.fochx.2026.104548 (PMC13671372; doi:10.1016/j.fochx.2026.104548)
Supplement: Supplementary file 9 [file mmc9.docx]

**Integrated multi-omics reveals lipid remodeling underlying population- and stage-associated aroma variation in chicken egg yolks**

Cui Ma ^a,b^, Jinsong Pi ^a,b^, Ailuan Pan ^a,b^, Yaru Chen ^a,b^, Ming Fu ^a,b^, Tao Huang ^a,b^, Hao Zhang ^a,b^, Yan Wu ^a,b*^

^a^ Institute of Animal Science and Veterinary Medicine, Hubei Academy of Agricultural Sciences, Wuhan, 430064, China.

^b^ Key Laboratory of Animal Embryo Engineering and Molecular Breeding of Hubei Province, Wuhan, 430064, China.

* Corresponding Author: Yan Wu, E-mail: [wuyanwh@163.com](mailto:wuyanwh@163.com)

**Figure legends**

**Figure S1 Permutation-test validation of PLS-DA models for egg yolk volatile profiles.** Permutation-test results (200 permutations) for PLS-DA models comparing (A) F31, FC, FH, and FA; (B) G31, GC, GH, and GA; (C) G31 vs. F31; (D) GC vs. FC; (E) GH vs. FH; and (F) GA vs. FA. The original model R²Y and Q² values are indicated together with the corresponding permutation-based P values. R²Y represents model fit for the response variable, whereas Q² represents cross-validated predictive performance. Detailed model-validation statistics are provided in Table S7.

**Figure S2 Representative chromatographic and reference mass-spectral information for the HS-SPME-GC–MS analysis of egg yolk volatile compounds.** (A) Representative total ion chromatogram (TIC) obtained from the HS-SPME-GC–MS analysis of an egg yolk sample. (B) Reference mass spectra of four representative odor-relevant aldehydes—2-undecenal, heptanal, nonanal, and (E)-2-octenal—used to support compound annotation based on the NIST/in-house database. The spectra shown in (B) are reference spectra used for annotation and were not acquired as full-scan mass spectra from the present samples, which were analyzed in selected ion monitoring (SIM) mode.

**Figure S3 Representative chromatographic and MRM detection profiles from the LC–MS/MS lipidomic analysis.** (A, B) Representative total ion chromatograms (TICs) obtained from the same representative egg yolk sample in negative- and positive-ion modes, respectively. (C, D) Corresponding MRM detection profiles obtained from the same representative sample in negative- and positive-ion modes, respectively. The lipidomic analysis was performed using an MRM-based acquisition strategy in which lipid species were monitored according to predefined precursor ion (Q1)–product ion (Q3) transitions and retention times (RTs). Individual full product-ion MS/MS spectra were not acquired during sample analysis.

**Figure S4 Additional lipidomic characterization of population- and stage-associated variation in egg yolks.** (A-B) PCA score plots of lipidomic profiles among chicken populations at the onset-of-lay and peak-laying stages. (C-F) Heatmaps of lipid species involved in major enriched pathways, including α-linolenic acid metabolism, glycerophospholipid metabolism, sphingolipid metabolism, and glycosylphosphatidylinositol (GPI)-anchor biosynthesis. (G) Hierarchical clustering heatmap of lipid species across major lipid subclasses.

**Figure S5 Stage-associated lipid remodeling across different chicken populations.** (A-F) Heatmaps of representative lipid species involved in significantly enriched KEGG pathways between the onset-of-lay and peak-laying stages in white broilers (FC vs. GC), high-yield layers (FH vs. GH), and local chickens (FA vs. GA).

**Figure S6 Representative chromatographic and MRM detection profiles from the widely targeted LC–MS/MS metabolomics analysis.** (A, B) Representative total ion chromatograms (TICs) obtained from the same representative egg yolk sample in negative- and positive-ion modes, respectively. (C, D) Corresponding MRM detection profiles obtained from the same representative sample in negative- and positive-ion modes, respectively. The MRM detection profiles illustrate the monitoring of predefined precursor/product ion transitions used for widely targeted metabolite detection.

**Figure S7 Stage-associated metabolomic changes in egg yolks across different chicken populations.** (A-F) PCA score plots of metabolic profiles among chicken populations and sampling stages. (G) Heatmap of differential metabolites between peak-laying and onset-of-lay local chickens (GA vs. FA). (H) Heatmap of representative metabolites involved in metabolic pathways associated with stage-related metabolic variation.

**Figure S8 Integrated metabolite–volatile correlation networks associated with egg yolk aroma variation.** (A) Pearson correlation networks between differential volatile compounds and amino acid-related metabolites involved in flavor-associated metabolic pathways. (B) Pearson correlation networks between differential volatile compounds and lipid-related metabolites involved in lipid remodeling. Correlation significance is indicated as **p* < 0.05, ***p* < 0.01, and ****p* < 0.001.

**Table S1 Laying rates of the four chicken populations at the two sampling stages.**

| **Population** | **Laying rate at 180 d (%)** | **Laying rate at 300 d (%)** |
| --- | --- | --- |
| Jianghan | 43.76 | 84.19 |
| Recessive White-feathered Broiler Breeders | 47.41 | 85.84 |
| Lohmann Brown | 56.75 | 91.13 |
| Chuqin No. 2 | 52.38 | 87.53 |

Note: The 180-d sampling point represented the onset-of-lay stage, with laying rates ranging from 43.76% to 56.75% across the four populations, whereas the 300-d sampling point represented the peak-laying stage, with laying rates ranging from 84.19% to 91.13%.

**Table S2 Ingredient composition and nutrient levels of the laying diet used at both sampling stages.**

| **Ingredients** | **Content (%)** |
| --- | --- |
| Corn | 61.00 |
| Soybean meal | 26.00 |
| Limestone | 8.00 |
| Premix ^1）^ | 5.00 |
| Total | 100.00 |
| **Nutrient levels** ^2）^ |  |
| Metabolizable energy (MJ/kg) | 11.30 |
| Crude protein (%) | 16.30 |
| Calcium (%) | 3.60 |
| Available phosphorus (%) | 0.34 |
| Lysine (%) | 0.75 |
| Methionine (%) | 0.34 |
| Threonine (%) | 0.55 |
| Methionine + cysteine (%) | 0.62 |

^1)^ The premix provided vitamins and trace minerals per kilogram of premix as follows: vitamin A, 175 KIU; vitamin D₃, 75 KIU; vitamin E, 750 mg; vitamin B₁, 50 mg; vitamin B₂, 100 mg; vitamin B₆, 75 mg; niacin, 600 mg; folic acid, 15 mg; Cu, 0.16 g; Fe, 1.2 g; Zn, 1.5 g; Mn, 1.6 g; I, 10 mg; Se, 4 mg.

^2)^ Nutrient levels were calculated. The same laying diet was provided to all four chicken populations at both sampling stages (180 and 300 d).
